# Polyphosphate drives bacterial heterochromatin formation

**DOI:** 10.1126/sciadv.abk0233

**Published:** 2021-12-22

**Authors:** Francois Beaufay, Haley M. Amemiya, Jian Guan, Joseph Basalla, Ben A. Meinen, Ziyuan Chen, Rishav Mitra, James C. A. Bardwell, Julie S. Biteen, Anthony G. Vecchiarelli, Lydia Freddolino, Ursula Jakob

**Affiliations:** 1 Department of Molecular, Cellular and Developmental Biology, University of Michigan, Ann Arbor, MI, USA.; 2 Cellular and Molecular Biology Program, Michigan Medicine, University of Michigan, Ann Arbor, MI, USA.; 3 Department of Computational medicine and Bioinformatics, Michigan Medicine, University of Michigan, Ann Arbor, MI, USA.; 4 Howard Hughes Medical Institute, University of Michigan, Ann Arbor, MI, USA.; 5 Biophysics Program, University of Michigan, Ann Arbor, MI, USA.; 6 Department of Chemistry, University of Michigan, Ann Arbor, MI, USA.; 7 Department of Biological Chemistry, Michigan Medicine, University of Michigan, Ann Arbor, MI, USA.

## Abstract

Heterochromatin is most often associated with eukaryotic organisms. Yet, bacteria also contain areas with densely protein-occupied chromatin that appear to silence gene expression. One nucleoid-associated silencing factor is the conserved protein Hfq. Although seemingly nonspecific in its DNA binding properties, Hfq is strongly enriched at AT-rich DNA regions, characteristic of prophages and mobile genetic elements. Here, we demonstrate that polyphosphate (polyP), an ancient and highly conserved polyanion, is essential for the site-specific DNA binding properties of Hfq in bacteria. Absence of polyP markedly alters the DNA binding profile of Hfq, causes unsolicited prophage and transposon mobilization, and increases mutagenesis rates and DNA damage–induced cell death. In vitro reconstitution of the system revealed that Hfq and polyP interact with AT-rich DNA sequences and form phase-separated condensates, a process that is mediated by the intrinsically disordered C-terminal extensions of Hfq. We propose that polyP serves as a newly identified driver of heterochromatin formation in bacteria.

## Teaser

Polyphosphate specifically cooperates with Hfq to silence prophages and mobile genetic elements in the bacterial chromosome.

## INTRODUCTION

Polyphosphate (polyP), a simple and energy-rich polyanion composed of phosphoanhydride- bonded phosphates, is an ancient biomolecule present in every organism tested so far (*1*). PolyP has a multitude of different functions, serving as a stress resistance and virulence factor in bacteria while acting as blood clotting factor and modulator of amyloidogenic processes in eukaryotes (*2*). The many activities ascribed to polyP have been attributed to its physicochemical properties, particularly as a metal chelator and energy source, and, as we recently found, its propensity to act as a chaperone-like protein-binding factor (*3*).

**Fig. 1.**
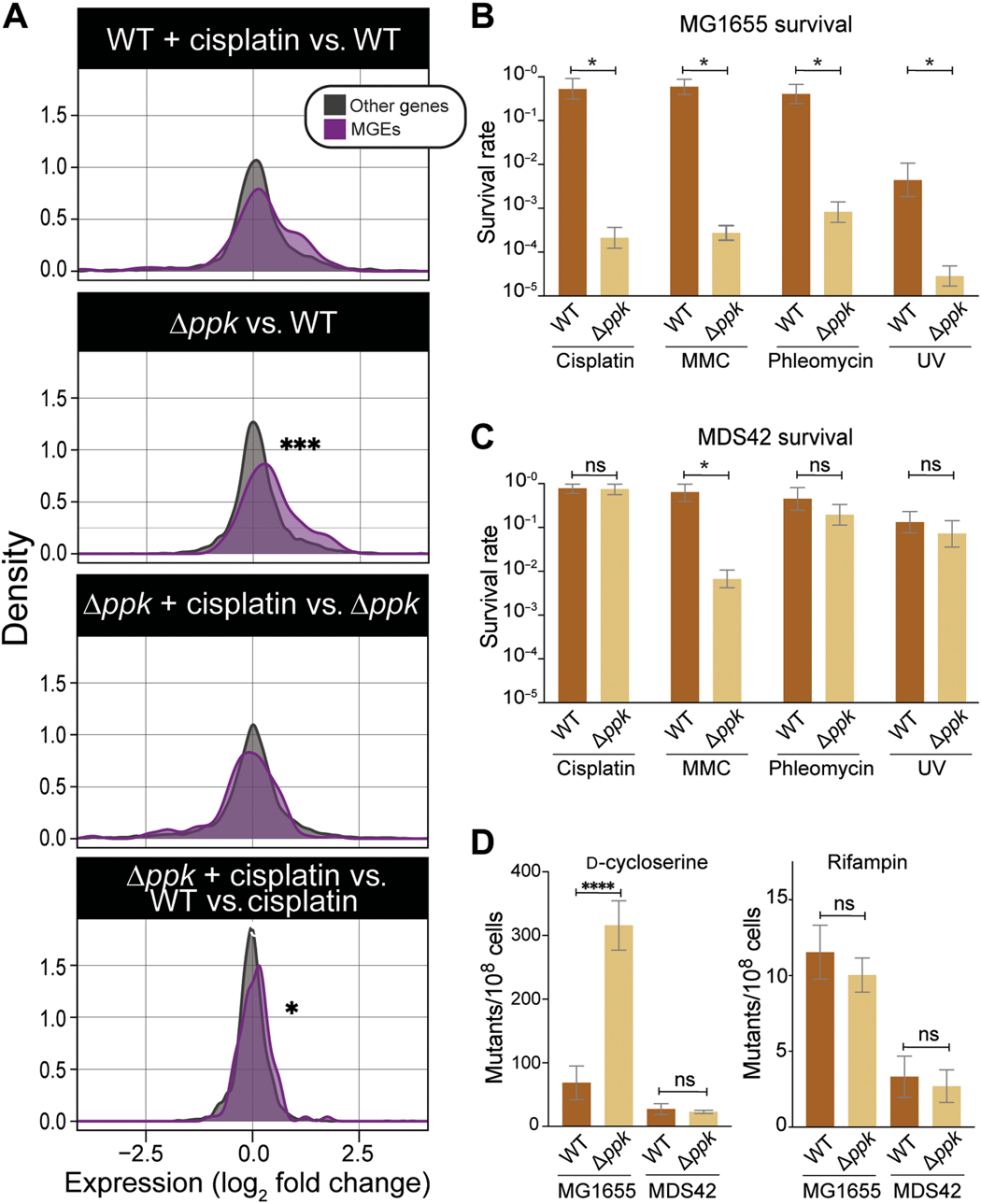
*Ppk* deletion increases sensitivity toward DNA-damaging agents through derepression of prophages and MGEs. (**A**) Density plots displaying the expression changes of MGEs (purple) and all other genes (gray) in WT or Δ*ppk* untreated or following a 15-min cisplatin exposure. Data are based on a published RNA-seq analysis (*4*). Significance stars represent *P* values from Mann-Whitney *U* test (*P < 0.05; ***P < 0.0005). (**B** and **C**) Survival of WT and Δ*ppk* cells in MG1655 (B) or MDS42 (C) background upon exposure to cisplatin (4 μg/ml), mitomycin C (MMC; 1 μg/ml), phleomycin (0.25 μg/ml), or ultraviolet (UV; 25 J/m^2^). Survival rates were scored after 16 to 24 hours growth at 37°C. Error bars show 95% credible intervals (see Materials and Methods for details); “*” indicates nonoverlapping 95% credible intervals for a particular comparison. (**D**) Mutagenesis rates for WT and Δ*ppk* cells in MG1655 or MDS42 background as determined by measuring colony-forming units on plates supplemented with either d-cycloserine or rifampin [*n* ≥ 3; ****P < 0.0005; ns, not significant; one-way analysis of variance (ANOVA)].

Recent studies showed that *Escherichia coli ppk* mutants (Δ*ppk*), which lack the polyP kinase (PPK) activity that is necessary to synthesize polyP, are sensitized toward the DNA cross-linking reagent cisplatin by altering the expression of iron homeostasis genes (*4*). Thus, polyP is important in counteracting cisplatin-elicited iron stress. Unexpectedly, however, RNA sequencing (RNA-seq) analysis revealed that Δ*ppk* mutants also show a pronounced enrichment of up-regulated RNAs coming from genomic regions containing mobile genetic elements and prophages (which we will collectively refer to as MGEs) (Fig. 1A and data S1) (*4*). Many of the genes in MGEs are known to be induced upon DNA damage, and their mobilization contributes to the lethal consequences of DNA damage stress (*5*). We found that the Δ*ppk* mutant strain not only displays a significantly augmented cisplatin-induced expression of MGEs relative to wild-type (WT) cells but also shows increased MGE expression levels in the absence of stress (Fig. 1A). These results suggest that lack of polyP leads to the derepression of MGEs and provide the first evidence that polyP might act in DNA damage control by preventing the mobilization of toxic MGEs.

## RESULTS

### PolyP suppresses transcription of MGEs

To investigate the idea that polyP is involved in the derepression of MGEs, we moved the *ppk* deletion into *E. coli* MDS42, a strain that lacks all previously identified MGEs (*6*). We reasoned that, if our conclusions were correct, then the removal of MGEs would make bacteria much less dependent on the presence of the *ppk* gene for survival following cisplatin exposure. Whereas deletion of *ppk* causes a significant increase in cisplatin sensitivity in the WT MG1655 background, it has no significant effect on cisplatin sensitivity in the MGE-deficient strain MSD42 over a wide range of cisplatin concentrations (Fig. 1, B and C, and fig. S1A). We observed a similar pattern in which the removal of the MGEs largely eliminates the DNA damage sensitization of *ppk* cells when we tested two other, unrelated DNA-damaging reagents, the DNA strand–breaking antibiotic phleomycin and ultraviolet (UV) light exposure, which causes pyrimidine dimers and other DNA lesions (Fig. 1, B and C) (*7*). In contrast, the removal of the MGEs only partially rescues the growth defect of the *ppk* deletion strain upon exposure to DNA cross-linking reagent mitomycin C (MMC), suggesting that, under those stress conditions, the polyP-mediated oxidative stress protection might play a more predominant role (*4*). Our results indicate that polyP likely protects against MGE derepression caused by a range of DNA-damaging conditions. Because polyP levels do not change upon treatment with DNA stressors such as cisplatin (*4*), we reasoned that the levels of polyP normally present within the cell are sufficient for polyP to fulfill this important role. To test whether polyP suppresses the mobilization of MGEs even in the absence of DNA-damaging conditions, we compared the growth of MG1655, MDS42, and their respective *ppk*-deleted strains in the presence of either d-cycloserine or rifampin. Whereas survival on d-cycloserine is acquired through a range of loss of function mutations in the amino acid transporter CycA (*8*), resistance to rifampin is almost entirely caused by specific point mutations in the rifampin-binding site of the β subunit of bacterial RNA polymerase (*9*). We thus hypothesized that derepression of MGEs should lead to a specific increase in insertional mutagenesis and hence a higher frequency in d-cycloserine–resistant (DSC^R^) cells without affecting the frequency of rifampin-resistant (rif^R^) cells. As shown in Fig. 1D, the absence of *ppk* did not alter the rif^R^ point mutation rate in either strain background. However, when we tested for acquisition of DSC^R^, we found that the lack of polyP synthesis increased the DSC^R^ mutation rate in MG1655 but not in the MGE-free strain MDS42. On the basis of these results, we concluded that the protective effect of polyP under DNA-damaging conditions is mediated by its ability to either directly or indirectly suppress the mobilization of MGEs—a hitherto unknown activity of polyP.

### PPK genetically interacts with the nucleoid-associated protein Hfq

**Fig. 2.**
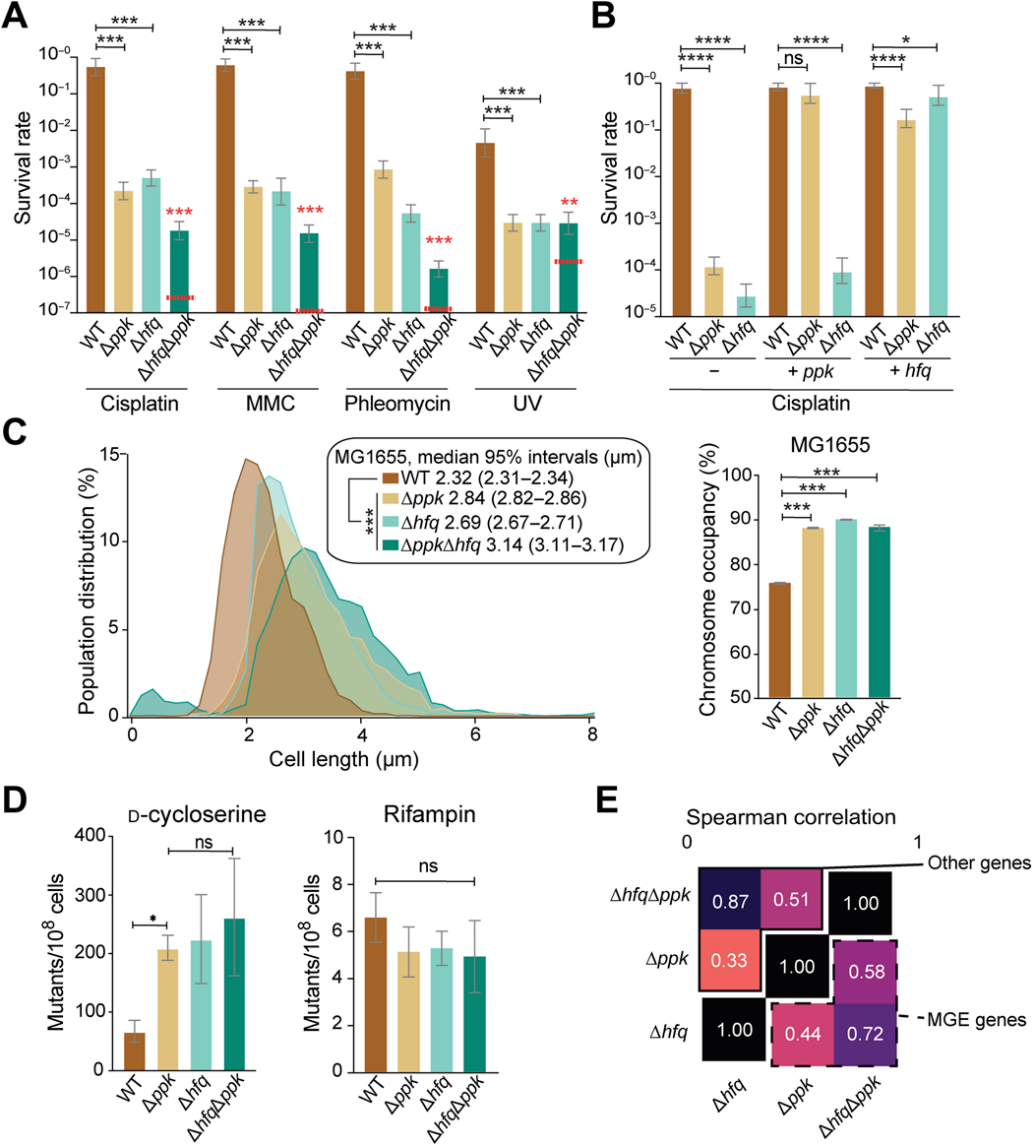
Hfq and polyP display epistatic relationship in vivo. (**A**) Survival assay of MG1655 *wt*, Δ*ppk*, Δ*hfq*, and Δ*hfq*Δ*ppk* upon exposure cisplatin (4 μg/ml), MMC (1 μg/ml), phleomycin (0.25 μg/ml), or UV (25 J/m^2^). Error bars show 95% credible intervals. Comparisons are scored on the basis of the posterior probability of a difference in parameters in the observed direction: *P > 0.95; ***P > 0.999. The red dashed lines indicate the predicted survival if the effects of the two single mutants were log additive. (**B**) Survival assay of WT, Δ*ppk*, or Δ*hfq* carrying either an empty plasmid (−) or a plasmid overexpressing *ppk* (*ppk*^+^) or *hfq* (*hfq*^+^) upon exposure to cisplatin (4 μg/ml). Error bars denote 95% credible intervals, with * markings based on the posterior probability of a difference in parameters in the observed direction: *P < 0.95; ****P < 0.9999. (**C**) Left: Cell size distribution of MG1655 *wt* (*n* = 8670*)*, Δ*ppk* (*n* = 8089), Δ*hfq* (*n* = 5875), or Δ*hfq*Δ*ppk* (*n* = 5954). Right: Associated space occupied by the nucleoid in each mutant under nonstress condition; confidence intervals are obtained on the basis of bootstrapping of the observed cell-level data, and significance calling is performed between each genotype and WT using a Wilcoxon rank sum test (***P < 0.005). (**D**) Mutant frequencies of WT, Δ*ppk*, Δ*hfq*, and Δ*hfq*Δ*ppk* strains upon growth on d-cycloserine or rifampin. (**E**) Gene expression analysis of MG1655 WT, Δ*ppk*, Δ*hfq*, and Δ*hfq*Δ*ppk*. Spearman correlation between phenotypes for “other genes” (continued box, upper triangle) or “MGE genes” (dashed box, lower triangle); all correlations are significant (*P* < 10^−5^ in all cases). RNA-seq differential expression analysis on all genotypes is compared to WT for cells grown in glucose minimal medium (corresponding to the non-cisplatin–treated condition in Fig. 1A).

Intriguing previous reports cite a potential role of polyP in DNA condensation both in *Pseudomonas aeruginosa* (*10*) and in cyanobacteria (*11*). These reports led us to consider the possibility that polyP might repress mobilization of MGEs by contributing to nucleoid condensation and chromosomal compaction. Comparative analysis of the large-scale nucleoid structure in MG1655 WT and Δ*ppk* strains reveals a significant expansion of the chromosome in bacteria lacking the *ppk* gene (fig. S1B). On the basis of the highly negatively charged character of polyP, however, we considered it very unlikely that polyP mediates chromosome compaction by directly interacting with DNA, another highly negatively charged molecule. Instead, we wondered whether polyP might act in concert with nucleoid-associated proteins, which are predominantly positively charged and which are responsible for compacting bacterial DNA (*12*). To genetically interrogate the interplay of nucleoid-associated proteins with polyP, we individually deleted the genes for six *E. coli* nucleoid-associated proteins (HupA, HupB, StpA, Hfq, Fis, and H-NS) in both MG1655 WT and Δ*ppk* cells and compared their growth in the absence and presence of cisplatin. We found that none of the individual deletion strains show a significant growth defect under baseline conditions (fig. S1C). However, when we combined the deletion of *fis* or *hns* with the *ppk* deletion, we found that the resulting double-mutant strains showed profound growth defects on agar (*fis/ppk* and *hns/ppk*) or in liquid medium (*hns/ppk*; fig. S1C); as the optical densities of cultures were standardized before spotting, the discrepant behavior of the *fis/ppk* double mutant on plates versus in liquid culture may reflect some combination of filamentous growth, low plating efficiency, or presence of large numbers of nonviable cells in the spotted culture. Possibly, because of their growth phenotypes, these double mutants are more resistant to cisplatin than the individual deletion strains (fig. S1D). While these results were consistent with a potential role of polyP in nucleoid organization, they were not necessarily indicative of a direct interaction between polyP and either Fis or H-NS. Instead, they imply that polyP might work in parallel to Fis and H-NS. We reasoned that, if polyP interactions were primarily mediated by Fis or H-NS, then one would expect that the single-mutant phenotype of the *fis* or *hns* deletions would be similar to that of the *ppk* deletion and that the double mutants would show a subadditive phenotype relative to the single mutants. We observed precisely this behavior, however, from an *E. coli* strain that lacks both *ppk* and *hfq*. Individually, the *ppk* and *hfq* deletion mutants show a similarly increased cisplatin sensitivity, while the *hfq/ppk* double mutant displays a much less severe cisplatin sensitivity than would be expected based on the individual effects of the single mutants (Fig. 2A). All the remaining deletions (i.e., *hupA*, *hupB*, and *stpA*), however, do not affect the cisplatin sensitivity of MG1655 and do not significantly alter the sensitivity of the *ppk* deletion strain (fig. S1D). We obtained equivalent results on the interaction of *hfq* and *ppk* when we compared the growth of WT, Δ*ppk*, Δ*hfq*, and Δ*hfq*Δ*ppk* strains in the presence of the three other DNA-damaging reagents tested (Fig. 2A). Under each stress condition, Δ*hfq* and Δ*ppk* strains display similarly increased sensitivities, while the Δ*hfq*Δ*ppk* mutant shows a less extreme sensitivity than would be expected based on the single-mutant phenotypes. This absence of a clear additive effect between *hfq* with *ppk* is indicative of an epistatic interaction, in which *hfq* and *ppk* contribute to DNA damage resistance along the same pathway.

To exclude the possibility that the observed effects are due to a polyP-dependent decrease in cellular Hfq levels, we monitored HfQ levels using MG1655 (WT) and Δ*ppk* strains, which express a fully functional *hfq-PAmCherry* construct that is driven by the native chromosomal promotor at the *hfq* locus (*13*). Western blot analysis using an anti-mCherry antibody showed that the steady-state levels of Hfq are very similar in the *ppk* deletion strain compared to the isogenic WT strain under nonstress conditions and increase in both strains upon exposure to cisplatin (fig. S1E). This result is consistent with RNA-seq data, which show that Hfq mRNA levels are up-regulated during cisplatin stress (WT versus WT exposed to cisplatin: log_2_ fold change +0.16, *q* value of 5.78 × 10^−10^; Δ*ppk* versus Δ*ppk* exposed to cisplatin: log_2_ fold change +0.37, *q* value of 0.07) and exclude the possibility that the observed effects of a *ppk* deletion are due to significant changes in the levels of Hfq protein. Plasmid complementation experiments furthermore demonstrated that the expression of a higher amount of Hfq can compensate for the absence of polyP in rescuing the DNA damage sensitivity phenotype, whereas excess PPK cannot compensate for the loss of Hfq (Fig. 2B and fig. S1F). These results led us to conclude that polyP acts upstream of Hfq and that a functional interaction such as binding between Hfq and polyP is more likely than a simple effect of the *ppk* deletion on Hfq levels.

### PolyP affects DNA-specific but not RNA-centric roles of Hfq

Hfq is an abundant hexameric protein with a multitude of biological roles in the cell (fig. S2A). One of the primary functions of Hfq is as an RNA chaperone, promoting the pairing of small RNAs and mRNA (state i) and assisting in ribosome biogenesis (state ii) (*14*, *15*). In another, less well-studied role, Hfq binds DNA and functions in chromosomal compaction (*16*, *17*); while some of these Hfq-DNA binding events have been shown to be mediated by interactions with nascent transcripts (state iii), others seem to reflect direct Hfq-DNA interactions (state iv) (*16*). Overall, 10 to 20% of the Hfq present in the cell is nucleoid-associated (*18*), corresponding to states iii and iv; with a total Hfq copy number of ~8300 molecules per cell in minimal medium (*19*), this leaves approximately 250 Hfq hexamers associated with the nucleoid during growth in minimal medium. To test whether polyP affects the RNA chaperone activity of Hfq, we analyzed the expression of six genes, whose expression levels have previously been shown to be regulated by small RNAs through their interaction with Hfq (*20*). Consistent with previous results, we found markedly different expression levels for all tested Hfq-controlled targets in the *hfq* deletion compared to WT MG1655 (fig. S2B). However, neither additional deletion of the *ppk* gene in the Δ*hfq* background nor the deletion of *ppk* in the WT background caused any differences in the expression of these genes compared to the parental strains (fig. S2B). These results argue against a role of polyP in the RNA chaperone activity of Hfq. Similarly, when we compared the cell shape and chromosome occupancy in these strains, we found that the nucleoids of Δ*hfq*, Δ*ppk*, and Δ*hfq*Δ*ppk* cells are all significantly elongated, with the double deletion showing roughly log-additive effects (Fig. 2C); thus, Hfq’s role in nucleoid compaction appears likewise to be polyP independent. From these results, we concluded the following: (i) polyP interacts with Hfq in MGE silencing but not in its RNA chaperone or large-scale nucleoid structuring activities; (ii) polyP most likely acts upstream of Hfq in terms of resistance to DNA damage; and (iii) on the basis of on our rescue experiments, polyP potentially functions by increasing the local concentration of Hfq on the DNA.

### PolyP and Hfq cooperate in silencing of MGEs

Protein occupancy profiling experiments have demonstrated the presence of heterochromatin-like domains termed extended protein occupancy domains (EPODs) across many regions of the *E. coli* chromosome, covering 10 to 15% of the genome under most conditions and showing enrichment on horizontally acquired regions such as MGEs (*21*). We have recently shown that, while many EPODs are composed of H-NS filaments, a substantial minority are not and likely represent regions that are silenced by assemblies of other nucleoid-associated proteins (*22*). We have recently shown that Hfq and Fis are required for silencing prophages across the genome and make up the main protein components of EPODs across many of these regions (*23*). Moreover, we found that loss of fis and hfq is synthetic lethal in a prophage-dependent manner (*23*). These findings and our present results, including the severe growth defect of the ΔfisΔppk strain (fig. S1C) and the epistatic interactions of *hfq* and *ppk*, suggested that polyP might cooperate with Hfq to silence MGEs. In support of this notion, we found that the mutagenesis rate of the Δ*hfq* deletion strain when tested on d-cycloserine is very similar to the mutagenesis rate of the Δ*ppk* deletion strain and comparable to mutagenesis rates seen in a strain that lacked both *hfq* and *ppk* (Fig. 2D). In contrast, no differences in the rifampin mutation rates are observed for any combination of *ppk* and *hfq* deletion relative to the parental strains (Fig. 2D). Last, RNA-seq analysis revealed significant similarity in expression changes between Δ*hfq* and Δ*ppk* strains both within MGEs and genome-wide (Fig. 2E). These results provided evidence that polyP might cooperate with Hfq to silence specific regions of the bacterial chromosome, particularly in regions containing MGEs and prophages.

### PolyP promotes Hfq specificity for AT-rich DNA sequences in vivo and in vitro

**Fig. 3.**
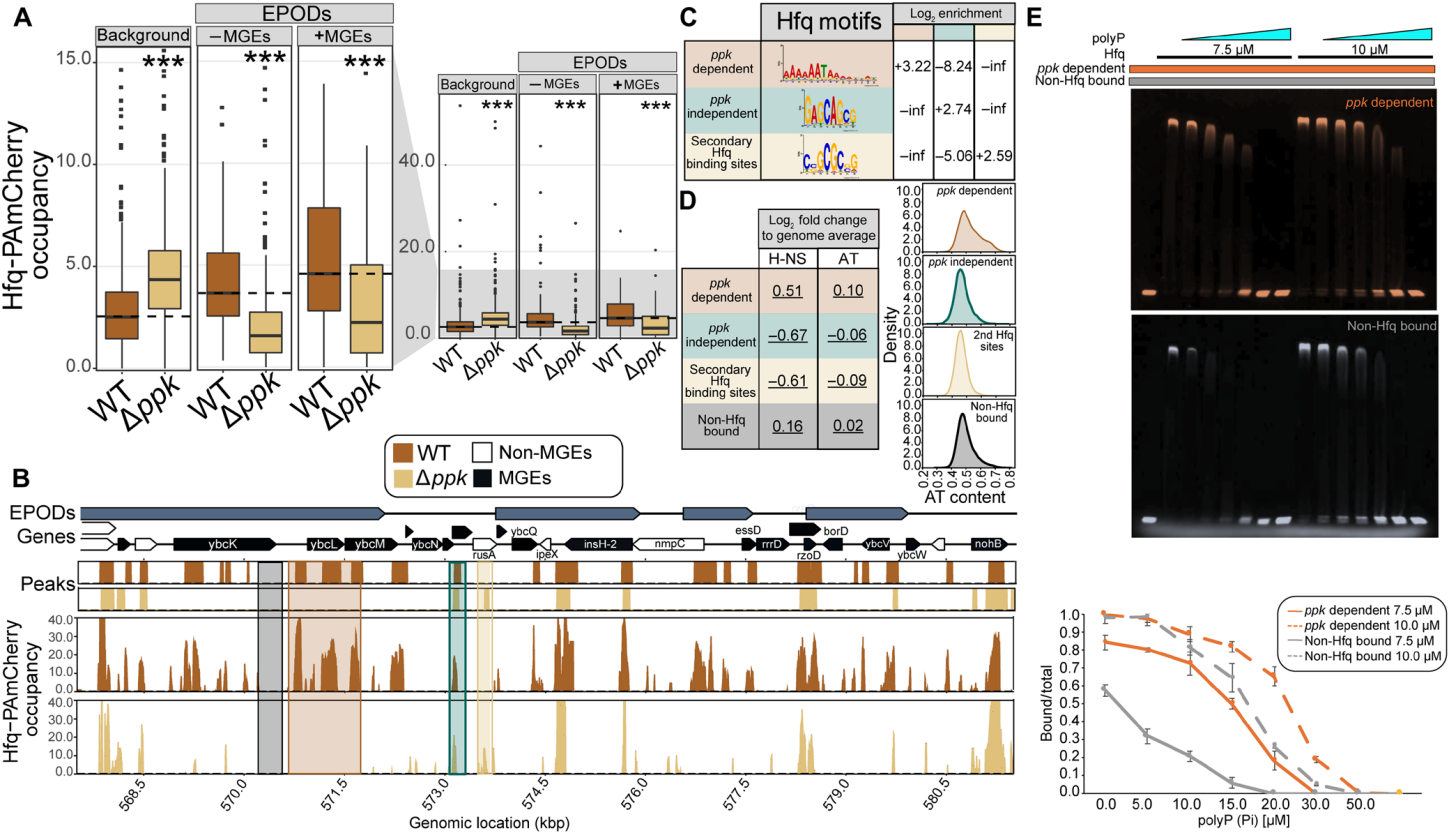
Loss of *ppk* alters DNA binding specificity of Hfq. (**A**) Average occupancy of Hfq-PAmCherry at chromosomal regions not covered by EPODs (background), at EPODs without MGEs (−MGEs), and at EPODs covering MGEs (+MGEs) (table S2). * indicates significance tested via a Wilcoxon rank sum test comparing Δ*ppk* versus WT for each condition with Benjamini-Hochberg corrections applied (***q < 0.0005). (**B**) Hfq-PAmCherry occupancy in MG1655 with *hfq*-PAmCherry (brown) and Δ*ppk* (gold) at selected prophage gene-containing region. Highlighted regions exemplify *ppk*-dependent (brown), *ppk*-independent (green), or secondary (gold) Hfq binding sites and unbound regions (black). kbp, kilo–base pair. (**C**) Top motif calculated from MEME-ChIP results for each peak category. The log_2_ motif enrichment was calculated across each category. Inf and −inf denote positive and negative infinity, respectively, and arise when there are no members of one of the classes being compared. (**D**) The 500-bp rolling mean for the binding of H-NS and the AT content of the genome was used to calculate the group-level means across each peak class. The log_2_ fold change was calculated in comparison with the overall average for the genome. Permutation test–based *P* values were calculated comparing each class versus the entire genome. The values underlined had *P* < 0.05. (**E**) Electrophoretic mobility shift assay (EMSA) of a mixture of 37.5 nM FAM-labeled AT-rich DNA (*ppk*-dependent, orange) and 37.5 nM Cy3-labeled GC-rich DNA (non-Hfq bound, gray) with Hfq and/or polyP present as indicated. PolyP_300_ concentrations are in ascending order: 5 10, 15, 20, 30, or 50 μM in Pi units. Gels were imaged and overlayed using ImageJ. Quantification of the % shifted DNA over total DNA was conducted in ImageJ. The average of three biological replicates is shown, with error bars representing SD.

To directly examine the impact of the *ppk* deletion on Hfq binding of bacterial DNA, we conducted chromatin immunoprecipitation sequencing (ChIP-seq) experiments on WT and Δ*ppk* strains expressing chromosomally encoded Hfq-PAmCherry; cells were treated with rifampin before cross-linking and treated with ribonuclease (RNase) during the ChIP workup, to ensure that only directly DNA-associated Hfq was detected (i.e., state iv in fig. S2A). To assess Hfq binding across the genome, we differentiated between three different DNA regions: all regions in the DNA that do not contain EPODs (i.e., nonheterochromatin regions), EPODs that encompass MGEs, and the rest of the existing EPODs (Fig. 3A and data S2). We found that the absence of *ppk* causes a specific and significant decrease of Hfq binding at EPODs and even more markedly at EPODs containing MGEs (Fig. 3A). In contrast, binding of Hfq to non-EPOD (nonheterochromatin) regions appears to increase in the absence of *ppk*, demonstrating that Hfq retains its ability to bind DNA in the absence of polyP, yet loses its specificity for DNA regions encoding prophages and mobile elements. Exemplary regions are shown in Fig. 3B and fig. S3A. These results help explain our earlier findings that lack of polyP causes the derepression of toxic mobile elements in an Hfq-dependent manner: In the absence of polyP, Hfq binding to MGEs is reduced. However, our data also raised the obvious question as to how the absence of polyP affects Hfq binding specifically across DNA regions enriched for MGEs. Comparative analysis of the DNA sequences that are bound by Hfq in a polyP-dependent versus polyP-independent manner, as well as the secondary Hfq binding sites that are only bound in the absence of polyP, revealed markedly different Hfq binding motifs (Fig. 3C and fig. S3B). We found that polyP-dependent Hfq motifs are highly AT-rich, while polyP-independent Hfq binding motifs have a much higher GC content (Fig. 3C and fig. S3B). In addition, we found that the polyP-dependent Hfq peaks had a clear enrichment for overlapping H-NS bound regions (Fig. 3D). Thus, the absence of polyP appears to alter the binding profile of Hfq, with a particular loss of Hfq tropism toward AT-rich regions containing MGEs. To test whether Hfq displays any polyP-dependent differences in DNA binding in vitro, we amplified two equally long DNA sequences, either encompassing a highly AT-enriched *ppk*-dependent binding site of Hfq or a GC-rich sequence encompassing a site that did not show Hfq binding in vivo (fig. S3C). Coincubation of the two differentially labeled DNA fragments with Hfq demonstrated that Hfq interacts with both fragments in vitro (Fig. 3E). Addition of increasing amounts of polyP, however, caused the preferential release of the GC-rich DNA sequence (Fig. 3E, gray) while maintaining binding to the AT-rich sequence (Fig. 3E, orange). These results support our in vivo findings that Hfq favors AT-rich regions in the presence of polyP.

### PolyP, Hfq, and DNA form high–molecular weight assemblies in vitro

**Fig. 4.**
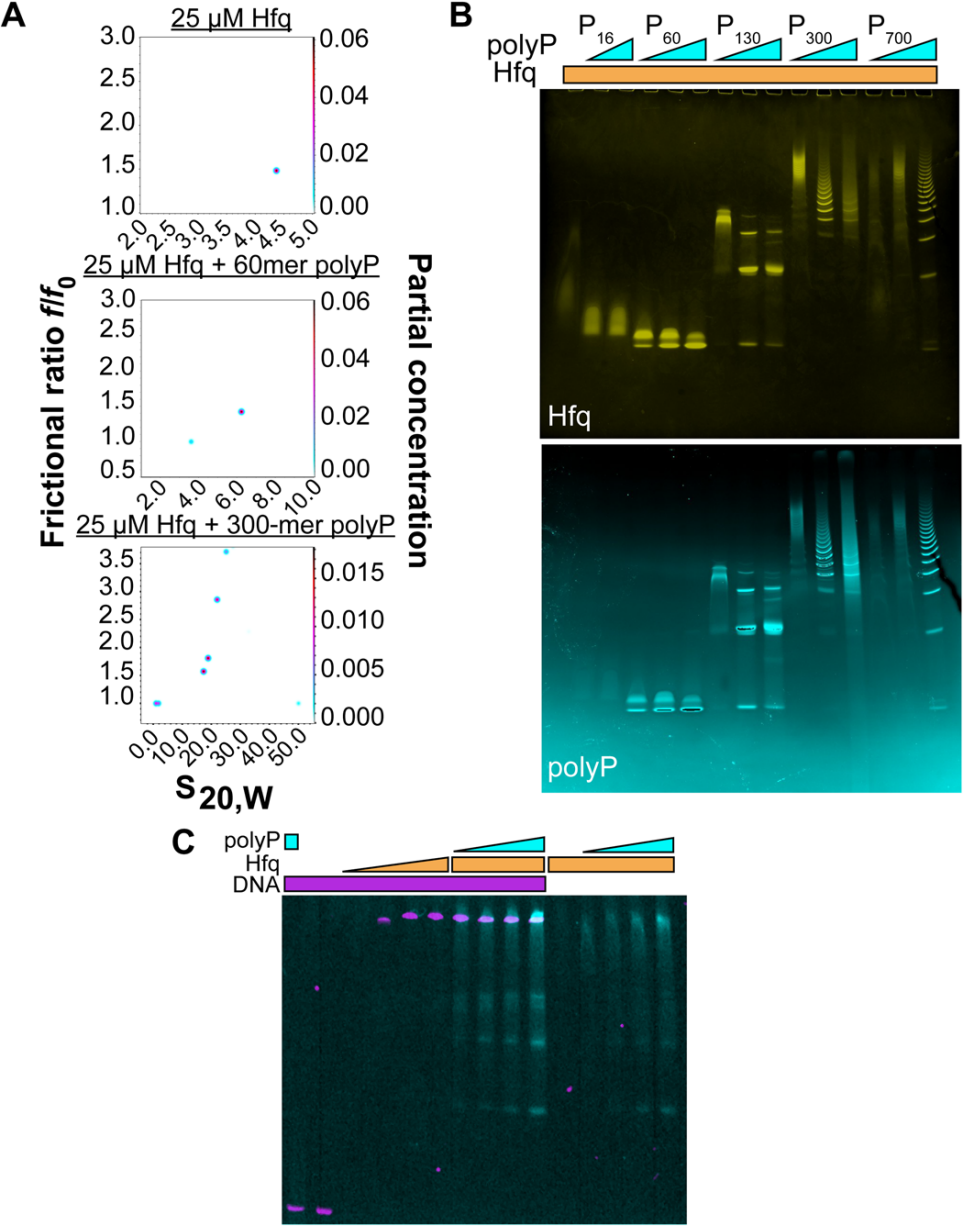
In vitro interactions of Hfq, polyP, and DNA. (**A**) Two-dimensional analysis plots of frictional ratio *f*/*f*_0_ versus sedimentation coefficient *S* for 25 μM Hfq ± 250 μM polyP_60_ or polyP_300_. The color in the *z* dimension represents the abundance of each species according to the partial concentration as indicated in the right *y* axis. (**B**) Mobility of Hfq (25 μM) on native gels without (lane 1) or with 65, 125, or 250 μM of various polyP chain lengths. PolyP_16_ was used at 125 and 250 μM only (lanes 2 and 3). Gels were stained with 4′,6-diamidino-2-phenylindole (DAPI) to visualize polyP (cyan) followed by Coomassie staining to visualize Hfq (yellow). (**C**) Overlay of EMSA using FAM-labeled *ybck*-DNA (75 nM) in the presence of 30 μM Alexa Fluor 647 (AF647)–polyP_300_ (lane 1); in the absence of additives (lane 2); or in the presence of 5, 10, 17.5, or 25 μM Hfq (lanes 3 to 6). AF647-polyP_300_ (in Pi units) was added at 10, 15, 20, or 30 μM to 25 μM Hfq and DNA (lanes 7 to 10) or 25 μM Hfq only (*11*–*15*). Gels were stained with Coomassie to detect Hfq (fig. S4B).

Combining our in vivo findings as well as published evidence for the activity of polyP in supporting protein assembly (*3*), we contemplated the possibility that polyP affects the activity of Hfq hexamers by promoting their association into larger oligomeric structures (*13*, *24*). Such oligomerization not only would increase the local concentration of Hfq at the vicinity of DNA but also could potentially provide the mechanical support for Hfq to silence select chromatin regions. We found that while purified Hfq (theoretical weight of monomer, 11 kDa) sediments in analytical ultracentrifugation experiments as a homogeneous hexamer with a sedimentation coefficient of 4.4*S* [estimated molecular weight (MW) of 70 kDa], the presence of a 10-fold molar excess (in P_i_ units) of polyP_60_ (MW, ~3.5 kDa) leads to the formation of a much larger 6.2*S* particle (Fig. 4A). Addition of the 300-mer polyP_300_ caused the formation of multiple large and heterogeneous complexes with sedimentation coefficients up to 30*S* (Fig. 4A). The increase in frictional ratios *f*/*f*_0_ for the larger complexes indicated elongated complexes that could possibly combine multiple polyP and Hfq molecules. These results provided first in vitro evidence that polyP and Hfq physically interact. This interaction was confirmed by native gels, which showed that Hfq and various length polyP chains form discrete bands when incubated together before electrophoresis (Fig. 4B). Yet, individually, both polyP and Hfq migrate as unfocused smears (fig. S4A). Mixing the *ybcK* gene sequence, shown by ChIP-seq to bind Hfq in a polyP-dependent manner in vivo (fig. S3A) with polyP and Hfq at roughly equimolar concentrations, results in comigration of all three components in native gels (Fig. 4C and fig. S4B). Further increase in the polyP_300_ to DNA and Hfq ratio, however, causes the formation of the previously observed range of higher MW assemblies, concomitant with the dissociation of Hfq from the DNA (fig. S4C). We concluded from these results that Hfq interacts with both polyP and specific DNA sequences to form high-MW assemblies. Increasing levels of polyP eventually compete with DNA binding to Hfq potentially through the formation of DNA-binding incompetent storage forms of Hfq or because of the direct occlusion of the DNA binding sites by polyP. In contrast, titrating increasing concentrations of polyP to a complex formed between Hfq and the mRNA essD, a known client of Hfq (*25*), did not cause the release of RNA even at polyP concentrations that converted most of Hfq into high-MW assemblies (fig. S4D). These results supported our in vivo findings, which demonstrated that the absence of polyP does not substantively affect the interactions between Hfq and RNA in bacteria.

### Hfq, polyphosphate, and DNA form three-component liquid droplets

**Fig. 5.**
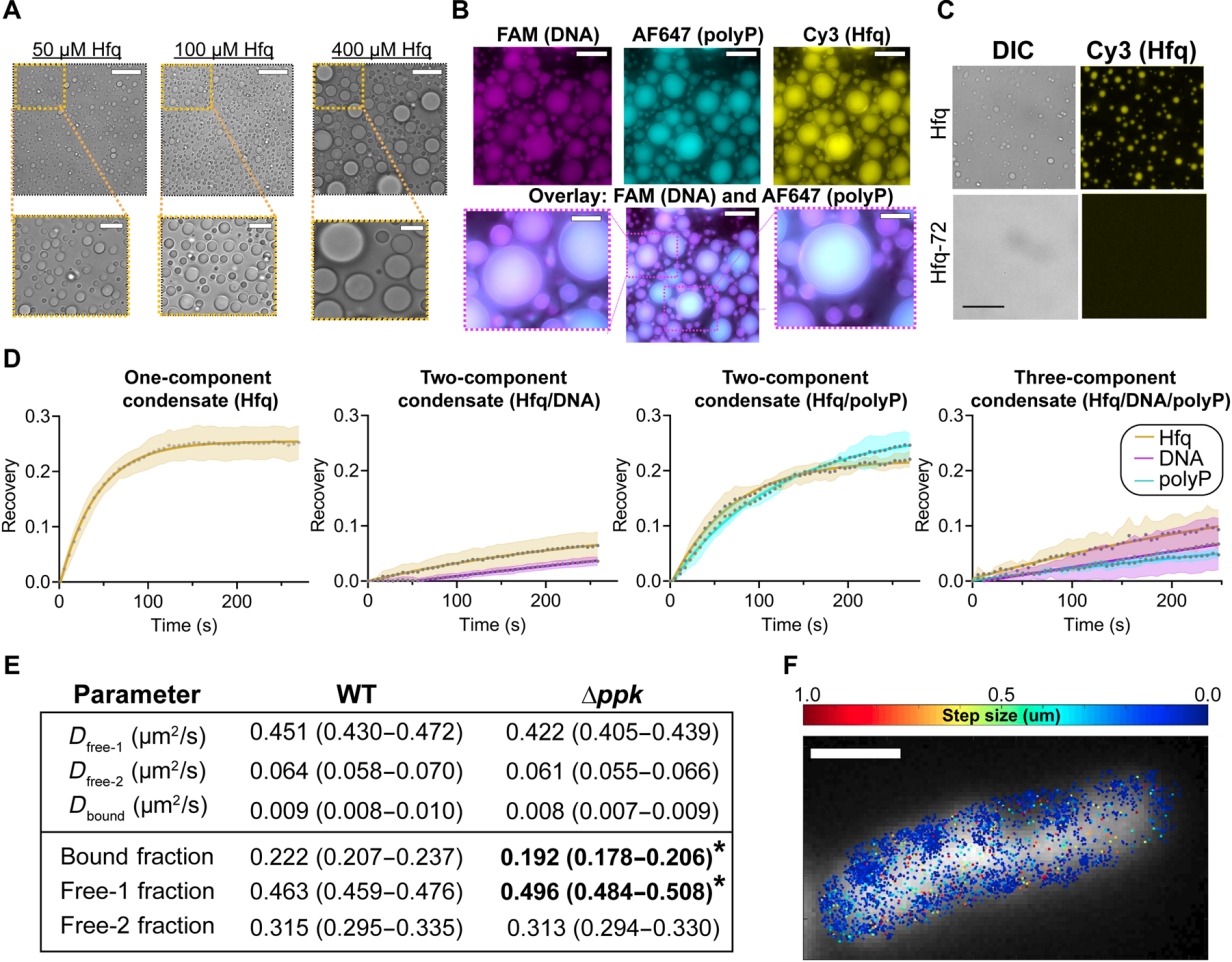
Hfq, polyP, and DNA form three-component condensates. (**A**) Differential interference contrast (DIC) images of purified *E. coli* Hfq upon dilution into low-salt buffer. (**B**) Fluorescence images of Hfq (100 μM) in the presence of 100 nM FAM-labeled DNA (magenta) and/or 100 μM AF647-labeled polyP_300_ (in Pi units) (cyan). Scale bars, 30 μm in full-size images and 10 μM in cropped images (D and E). (**C**) DIC and fluorescence images of 25 μM Hfq or 25 μM Hfq-72. Cy3-labeled Hfq was spiked in at 1/75 concentration for visualization of the droplets. Scale bars, 20 μm. (**D**) FRAP analysis of Hfq (yellow) with or without DNA (magenta) and/or polyP (cyan). Droplets were half-bleached, and normalized recoveries were fit to a one-phase association curve to obtain the recovery half-times: Hfq alone (Hfq *t*_1/2_ = 29 ± 3 s); Hfq + DNA (Hfq *t*_1/2_ = 194 ± 31 s, DNA *t*_1/2_ = 806 ± 104 s); Hfq + polyP (Hfq *t*_1/2_ = 47 ± 4 s, polyP *t*_1/2_ = 82 ± 9 s); and Hfq + DNA + polyP (Hfq *t*_1/2_ = 293 ± 185 s, DNA *t*_1/2_ = 18671 ± 9104 s; polyP *t*_1/2_ = 612 ± 388 s). Error represents SD from *n* ≥ 3 droplets per condition. (**E**) Fit values (with 95% confidence intervals in parentheses) for diffusion constants and state occupancies from Spot-On analysis of the Hfq single-molecule tracking data (the free-2 state occupancy reflects the balance of all occupancy and is thus estimated on the basis of the bound and free-1 occupancies, with a normal approximation used for the confidence interval for this parameter); * indicates a significant difference between genotypes assessed by nonoverlapping 95% confidence intervals. (**F**) Representative results of Hfq single-molecule tracking experiments showing the observed single-molecule displacements between individual Hfq snapshots (color scale), superimposed on the DAPI channel (gray scale). Scale bar, 1 μm.

Hfq contains intrinsically disordered, low-complexity regions, which are known for their propensity to undergo liquid-liquid phase separation (LLPS), particularly in the presence of polyanions (*26*). Moreover, we observed that solutions of purified Hfq become turbid as the salt concentration is dropped in a reversible manner, a hallmark of LLPS. These pieces of evidence led us to consider that Hfq, polyP, and DNA might undergo LLPS, which is an increasingly recognized mechanism for chromatin organization in eukaryotes (*27*). Microscopic analysis revealed that dilutions of Hfq into low-salt buffer yielded uniform, spherical liquid droplets that flow and fuse with one another and readily dissolve when transferred back into high-salt buffer (Fig. 5A and movies S1 to S7). Turbidity measurements confirmed that Hfq undergoes LLPS at concentrations well below its cellular concentration of ~90 μM (calculated from 55,000 molecules in an *E. coli* cell of 1 fl in volume) (*28*) and at physiologically relevant salt concentrations even without the addition of crowding agents (fig. S4E). Addition of either DNA or polyP leads to the sequestration of both polymers into Hfq-containing liquid droplets (Fig. 5B), while neither DNA nor polyP phase-separates in the absence of Hfq or under high-salt conditions (fig. S4F). Moreover, we were unable to detect any significant accumulation of essD RNA in the liquid droplets, indicating that the sequestration of polyP and DNA is specific (fig. S4G). We also found that the addition of polyP or DNA increases the turbidity of Hfq solutions (fig. S4, H and I) and that polyP slightly lowers the saturation concentration for liquid-droplet formation of Hfq (fig. S4J). Increasing the polyP levels further, however, dissolves the liquid droplets, concomitant with the formation of the previously observed high-MW Hfq assemblies (fig. S4I).

### Hfq’s C terminus mediates polyP interactions and drives LLPS

To provide additional insight into the nature of the Hfq-polyP-DNA interactions that we observed, we decided to use a truncation mutant of Hfq, which lacks the C-terminal extensions thought to be involved in the binding of AT-rich DNA sequences (*29*) and potentially in in vivo nucleoid association (*30*). Truncation of the C-terminal extensions of Hfq (i.e., Hfq-72) not only significantly reduces DNA binding and alters the interactions of Hfq with polyP (fig. S4K) but also abolishes the ability of Hfq to phase-transition. Dilution of purified Hfq-72 into low-salt buffer does not lead to the formation of any detectable droplets at concentrations where full-length Hfq readily phase-separates (Fig. 5C). Turbidity measurements revealed that the addition of neither polyP nor DNA causes any significant increase in the turbidity of Hfq-72 upon dilution into low-salt buffer (fig. S4L). In summary, these results strongly suggest that Hfq, DNA, and polyP form a three-component phase-separated biomolecular condensate likely interacting via the intrinsically disordered C termini of Hfq. We further assessed the in vivo relevance of these findings by testing whether overexpression of the Hfq-72 construct could complement loss of chromosomal Hfq in our cisplatin survival assay and found that the Hfq-72 showed only partial rescue, whereas full-length Hfq showed robust rescue (fig. S4M). These results provide additional in vivo evidence for the involvement of Hfq in silencing MGEs through its binding to AT-rich DNA, its ability to phase-separate with polyP, or, most likely, a combination thereof.

### PolyP alters the properties of Hfq liquid droplets

To test whether and how polyP and/or DNA affect or regulate the structure of Hfq condensates, we conducted fluorescence recovery after photobleaching (FRAP) experiments, monitoring recovery of all three components upon their bleaching. For these experiments, we visually tracked all three components by supplementing native Hfq with Cy3-labeled Hfq-S65C [which has been shown to behave like WT protein both in vivo and in vitro (*31*)] and used fluorescein amidites (FAM) labeled *ybzK*-DNA and Alexa Fluor 647 (AF647)–labeled polyP_300_. In the absence of any polyanions, we found that the fluorescence of Hfq droplets, when photobleached over half of the droplet (i.e., half-FRAP), recover with a *t*_1/2_ of 29 ± 6 s (Fig. 5D). Recovery of mixed polyP/ Hfq occurs at a slightly slower rate than the recovery rate of Hfq alone, fully consistent with the formation of higher-MW polyP-Hfq complexes. The presence of DNA further slows Hfq recovery (Fig. 5D). Yet, the three-component condensates show the slowest recovery rates for all three components, consistent with the formation of a ternary complex of Hfq hexamers, polyP, and DNA.

### PolyP increases the proportion of slowly diffusing Hfq complexes in vivo

To examine the effects of polyP on Hfq dynamics in vivo, we performed single-molecule fluorescence tracking experiments using strains that express Hfq-PAmCherry from the native genomic locus. This fusion protein was generated and characterized in (*32*), showing no notable effects on Hfq activity; for our purposes, the *hfq-pamcherry* allele was transduced into our WT and Δ*ppk* backgrounds. Analysis of the Hfq diffusion constants using SpotOn (*33*) to identify the different diffusive states and their abundances revealed three distinct Hfq states in both strain backgrounds (Fig. 5, E and F). We refer to these states as free-1, free-2, and bound, in descending order of diffusion constants. While the diffusion constants of the three states are very similar in WT and Δ*ppk* cells, the population of the bound state is significantly lower in Δ*ppk* cells than in WT, indicating that the loss of polyP reduces the number of slowly diffusing Hfq complexes in vivo (Fig. 5E). We were unable to resolve a clearly distinct state corresponding to chromatin-bound or phase-separated Hfq in vivo, likely because the vast majority of Hfq is involved in RNA binding/metabolism rather than DNA binding. Nevertheless, these results are consistent with the formation of higher-MW liquid-liquid phase transition complexes that incorporate Hfq, DNA, and polyP and lead us to conclude that we have found a hitherto unknown role of polyP in bacterial heterochromatin formation.

## DISCUSSION

### PolyP promotes preferential binding of Hfq to AT-rich regions causing gene silencing

Bacterial evolution is greatly accelerated through horizontal gene transfer, which significantly contributes to the genetic diversity within bacterial species and plays a major role in pathogenicity and antibiotic resistance (*34*). However, many genetic elements, particularly prophages and insertion sequence (IS) elements, come with their own specific hazards, as they can readily cause mutations and cell death if mobilized. Because these hazards can pass from a donor to a recipient cell during any horizontal gene transfer process, they would tend to limit horizontal gene transfer events. To combat these risks, the *E. coli* protein H-NS and its homologs act as xenogeneic silencers, with a propensity to form silencing protein assemblies on horizontally acquired AT-rich DNA sequences, which are known for their high intrinsic curvature and flexibility (*35*, *36*). Similar xenogeneic silencing systems play important roles in a wide range of bacterial species (*37*). We now find that Hfq, facilitated by polyP, appears to be part of a separate xenogeneic silencing system. The question thus arises as to how seemingly nonspecific DNA binding proteins like Hfq (*38*) are able to select these specific regions in the DNA and form heterochromatin-like structures. Here, we demonstrate that the interaction of Hfq with AT-rich prophage/MGE regions is directly mediated by the polyanion polyP. We found that lack of polyP leads to the dispersal of Hfq specifically from AT-rich and H-NS–enriched MGEs, causing derepression of transcription in those regions. Unexpectedly, however, the absence of polyP did not cause a general loss of Hfq-DNA binding but instead changed the DNA binding profile of Hfq to show a relative preference for GC-rich regions while vanishing from MGEs. The effect of polyP on Hfq binding provides a simple and consistent explanation for the many unexpected similarities between *hfq* and *ppk* phenotypes, particularly in regard to how they affect chromosome structure, prophage-dependent increased stress sensitivity toward unrelated DNA-damaging reagents, and MGE expression profiles: polyP promotes the preferential binding of Hfq to AT-rich regions, leading to transcriptional silencing.

### PolyP promotes formation of phase-separated condensates by scaffolding Hfq

**Fig. 6.**
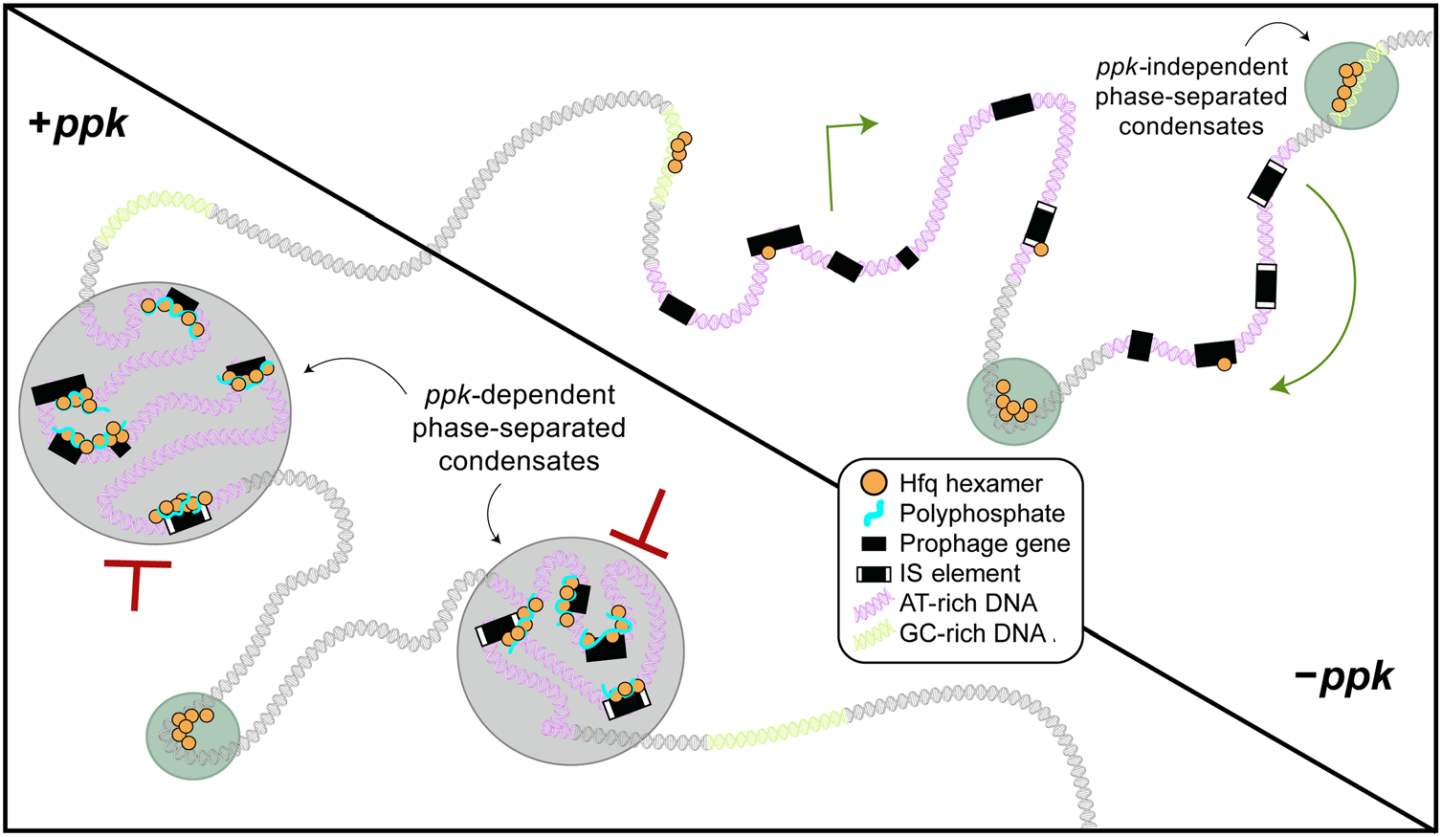
Working model for the formation of Hfq-polyP–based heterochromatin in *E. coli*. In WT *E. coli* (lower left quadrant), Hfq, polyP, and DNA work together to form bacterial heterochromatin by creating phase-separated droplets. PolyP drives the formation of these structures preferentially over AT-rich regions including prophages and MGEs, whose mobilization could damage the cell. The absence of polyP (top right quadrant) leads to the reorganization of the bacterial chromatin with an overall decrease in Hfq-heterochromatin complexes, and the relocation of Hfq from AT-rich to more GC-enrich motifs. This leads to the unsolicited derepression of toxic MGE sequences.

The apparent effect of polyP on Hfq binding also raised the intriguing question as to how polyP, a highly negatively charged polymer, could possibly enable the interaction of Hfq with another highly negatively charged polymer such as DNA, particularly in a nucleotide-specific (i.e., AT-rich versus GC-rich) manner. We have now shown that Hfq readily undergoes liquid-liquid phase transition in vitro, in a process that is further stimulated by the presence of DNA and polyP. Both polymers phase-transition with Hfq individually and together, forming discrete three-component condensates. Our FRAP experiments furthermore revealed that the fluorescence recovery in the three-component condensates is slower compared to droplets that are formed by Hfq alone. This behavior is likely due to the combined capacity of polyP and DNA to noncovalently assemble Hfq hexamers into higher-MW structures. Analysis of the Hfq-72 mutant variant revealed that the disordered C-terminal extensions of Hfq are crucial for the ability of Hfq to phase-transition, bind DNA, and interact with polyP, consistent with previous results that showed that the C termini of Hfq mediate the binding of Hfq to AT-rich DNA regions with high intrinsic curvature (*39*, *40*). In addition, we found that loss of polyP in vivo leads to the dissociation of Hfq from AT-rich regions, concomitant with a decrease in the fraction of Hfq present in a slowly diffusing and thus apparently complexed state. In contrast with all of our findings on Hfq-DNA associations, we have not been able to identify any strong effect of polyP on Hfq-RNA interactions: polyP appears not to directly compete with RNA for binding of Hfq in vitro, and the RNA chaperone activity of Hfq appears to be polyP independent in vivo. Combining our in vitro and in vivo data, we propose that Hfq has both a high-affinity and low-affinity binding site for polyP. Binding of polyP to the high-affinity binding site appears to promote the formation of trimeric Hfq-polyP-DNA complexes, drives Hfq toward binding of AT-rich DNA (including MGEs), and stabilizes it in a more tightly bound, phase-separated state (Fig. 6). In the presence of higher concentrations of polyP (≥5:1 molar ratio of polyP in Pi units and Hfq monomers), however, polyP directly competes with DNA and causes the formation of high-MW Hfq complexes, which no longer bind DNA and no longer phase-separate. It is conceivable that direct interactions between polyP and the intrinsically disordered C termini serve to noncovalently cross-link Hfq hexamers in a DNA binding–incompetent high-MW storage form.

### Hfq-polyP complexes: A previously unknown element in bacterial chromosomal structure

The LLPS undergone by Hfq likely arises because of weak associations between the disordered C-terminal tails of Hfq hexamers, with polyP acting as an additional intermediate increasing Hfq association by binding of multiple Hfq hexamers to a single polyP. PolyP thus appears to be doubly effective in promoting silencing of MGEs, by both directing preferential binding of Hfq to those regions and promoting the formation of phase-separated condensates, which likely render genes inaccessible to RNA polymerase. These results are reminiscent of the heterochromatin-like protein HP-1, which plays a major role in the formation of eukaryotic heterochromatin. Like Hfq, HP-1 undergoes phase transition with DNA by forming high-MW structures to permit heterochromatinization and gene silencing (*41*, *42*). As observed in our plasmid-based complementation experiments, in the absence of polyP, substantially higher amounts of Hfq are needed to achieve similar levels of prophage silencing, and, in all likelihood, more nonspecific Hfq binding occurs throughout the chromosome under such conditions. The Hfq-polyP complex appears to represent a fundamental and important element in bacterial chromosomal structure. Its role appears to complement that of other mechanisms of heterochromatin-like regulation of gene expression such as H-NS filament formation. The mechanism that we have identified here by which LLPS of heterochromatin-like domains silences potentially harmful genetic elements in *E. coli* may represent the first example of a broader paradigm for gene silencing in bacteria.

## MATERIALS AND METHODS

### Bacterial strains and growth conditions

All strains, plasmids, and oligonucleotides used in this study are listed in table S1. Null mutations in *E. coli* MG1655 (*43*) and MDS42 (*8*) were constructed as previously described (*44*). WT and mutant *E. coli* strains were grown at 37°C in either lysogeny broth (LB, Thermo Fisher Scientific) or MOPS minimal medium (Teknova) containing 0.2% (w/v) glucose and 1.32 mM KH_2_PO_4_ (MOPS-G). To conduct mutagen resistance tests on plates, M9 minimal medium containing 0.2% (w/v) glucose (M9-G) was supplemented with 1.5% (w/v) agar.

### Survival assays upon treatment with DNA-damaging reagents

Survival assays were performed as described previously (*4*). To determine the bacterial survival upon treatment with cisplatin (Sigma-Aldrich), MMC (Sigma-Aldrich), or phleomycin (Sigma-Aldrich), the various strains were cultivated in MOPS-G medium until an optical density at 600 nm (OD_600_) of 0.5 was reached. The bacteria were then 10-fold serially diluted, plated onto M9-G plates containing the indicated concentrations of the respective mutagens, and incubated overnight at 37°C. To determine survival upon UV treatment, cells were grown in LB medium at 37°C until an OD_600_ of 0.5 was reached. The bacteria were washed in ice-cold water and UV-C–irradiated (25 J/m^2^) using a spectrolinker XL-1500 UV cross-linker. All radiation experiments were performed on ice. Following irradiation, cells were 10× serial-diluted, plated onto LB agar plates, and incubated overnight at 37°C. The colony-forming units (CFUs) were scored after the incubation. Survival assays were analyzed by fitting a separate Bayesian model to each genotype-condition combination, in which the observed cell counts were treated as Poisson random variables arising because of the (known) dilution of each counted spot/plate, the (unknown) log_10_-scaled number of CFUs per milliliter υ_i_ initially present for a particular replicate experiment *i*, and the log_10_-fold change between the treated and untreated cells δ, taken to be constant for any particular genotype-stress combination. Thus, the experimentally observed counts for a particular replicate *i* were modeled as

$${\text{Untreated count}}_{i}\sim \text{Poisson}(\text{power}(10,\upsilon_{i}+\varphi_{i,\text{untreated}}))$$

$${\text{Treated count}}_{i}\sim \text{Poisson}(\text{power}(10,\upsilon_{i}+\delta +\varphi_{i,\text{treated}}))$$

where φ_i_ indicates the log_10_-fold dilution of the cells counted in a particular experiment and power(*x*,*y*) denotes *x^y^*. We used a Uniform(0,15) prior for the υ parameters and Uniform(−10,10) priors for the δ parameters. Models were fitted using pymc3 (*45*) and assessed for convergence based on the Rhat metric and inspection of the posterior predictive distributions. Posterior distributions of the δ parameters were used for subsequent analysis and significance calling as described in the text.

### Mutagenesis assays

To determine the rate and spectrum of mutations, bacteria resistant to d-cycloserine or rifampin were selected. For each strain, four independent cultures were diluted into MOPS-G at a density of 10^3^ cells/ml and grown for 24 hours at 37°C. Aliquots from each culture were serial-diluted and plated either onto MOPS-G plates containing d-cycloserine (Sigma-Aldrich) at 2× minimum inhibitory concentration (i.e., WT, 4 μg/ml; Δ*ppk*, 2 μg/ml) or onto LB agar plates containing rifampin (200 μg/ml) (Sigma-Aldrich). Plates were incubated at 37°C for 30 hours or overnight, respectively, before CFUs were scored. The total number of cells in each sample was determined by spreading dilutions onto nonselective LB plates. The mutagenesis rate was calculated as the ratio of CFUs on selective plates over the total number of cells in each sample (*6*).

### Bacterial nucleoid analysis

For fluorescent microscopy, exponentially growing bacterial strains cultivated in MOPS-G medium were imaged after immobilization on 1% (w/v) agarose pads. Bacterial nucleoids were stained with 4′,6-diamidino-2-phenylindole (DAPI) (1 μg/ml; Thermo Fisher Scientific) for 15 min and then washed in MOPS-G before immobilization. Fluorescence microscopy images were taken using a Zeiss Axiobserver.Z1 microscope equipped with an ORCA-Flash 4.0 complementary metal-oxide semiconductor (CMOS) camera and filter set 00. Images were processed using the MicrobeJ (*46*) suite for ImageJ. Chromosome occupancy of each cell was measured as the ratio between the DAPI area (fluorescent signal) and the total cell area (phase contrast). To analyze cells by electron microscopy, cells were grown in MOPS-G until mid-log phase was reached. Then, the cells were preliminary fixed using 2.5% (w/v) glutaraldehyde diluted in 0.1 M sodium cacodylate (pH 7.2) and stored at 4°C. Fixed cells were washed in 0.1 M sodium cacodylate buffer and postfixed in 1% (w/v) osmium tetroxide (OsO_4_) in 0.1 M sodium cacodylate (pH 7.2) for 1 hour at room temperature (*47*). Samples were then dehydrated through a series of washes with increasing concentrations of acetone (from 30 to 100%) and embedded in an epoxy resin (Epon). The resins were sliced by an ultramicrotome into thin (approximately 50 to 60 nm) sections and applied to glow-discharged carbon-coated grids (Pelco). The grids were stained with 2% (w/v) uranyl acetate (Electron Microscopy Sciences) for 1 min, washed with a drop of distilled water, blotted, and air-dried. Images were taken at 80 kV on a tecnai 10 transmission electron microscope with a Gatan 967 slow-scan, cooled US 4000 charge-coupled device (CCD) camera.

### ß-Galactosidase assay

To monitor the effect of polyP on the RNA chaperone activity of Hfq in vivo, we used previously established reporter constructs (*20*). Overnight cultures expressing the reporter constructs were diluted and grown until mid-log phase, and β-galactosidase fusion reporters were induced with 0.1% (w/v) arabinose for 1 hour before assaying. All in vivo β-galactosidase assays were performed as previously described (*48*).

### Cell growth and harvest for RNA-seq and Hfq-PAmCherry ChIP

Cells (WT, WT-Hfq-PAmCherry, and Δ*ppk*-Hfq-PAmCherry; two biological replicates for each genotype) were streaked onto an LB plate from cryogenic storage and grown at 37°C. Individual colonies were used to inoculate MOPS-G medium, and cultures were incubated at 37°C and shaking at 200 rpm. After overnight growth, cells were back-diluted to an OD_600_ of 0.003 and grown to a target OD_600_ of 0.2. Once the target OD was reached, 2.5 ml of the culture was mixed with 5 ml of RNAprotect (Qiagen, catalog no. 76506), vortexed, and incubated at room temperature for 5 min. The tubes were spun at 4°C for 10 min at 5000*g* in a fixed-angle rotor, shock-frozen in a dry-ice ethanol bath, and stored at −80°C. RNA isolation and sequence preparation is described below. The remaining culture was treated with rifampicin (150 μg/ml) for 10 min at 37°C and 200 rpm. The cultures were then mixed with concentrated formaldehyde/sodium phosphate (pH 7.4) buffer in Falcon tubes to achieve a final volume of 10 mM NaPO_4_ and 1% (v/v) formaldehyde. Tubes were placed into a shaker for 5 min at room temperature. Excess glycine (final concentration, 0.333 M) was added to quench the cross-linker, and the samples were incubated for 10 min at room temperature with shaking. The tubes were then placed on ice for 10 min and spun in a fixed-angle rotor for 4 min at 4°C and 5000*g*. After discarding the supernatant, the respective pellets were washed twice with 10 ml of ice-cold phosphate-buffered saline, dried, snap-frozen in a dry-ice ethanol bath, and stored at −80°C.

### Preparation of mCherry ChIP

Frozen pellets were resuspended in 600 μl of 1× lysis buffer [10 mM tris-HCl (pH 8.0) and 50 mM NaCl] with 1× protease inhibitors (cOmplete Mini, EDTA-free Protease Inhibitor; Roche) and Ready-Lyse (52.5 kU/ml; Lucigen) and incubated at 30°C for 15 min. Then, the tubes were placed on ice and sonicated with a Branson digital sonicator with 25% power, 10 s on, 10 s off, for a total of 30 s at 4°C. Tubes were kept in an ice water bath for the entirety of the sonication process. The sonicated lysates were then placed on ice and digested with 6 μl of RNase A (Thermo Fisher Scientific), 6 μl of deoxyribonuclease I (DNase I) (Thermo Fisher Scientific), 5.4 μl of 100 mM MnCl_2_, and 4.5 μl of 100 mM CaCl_2_, mixed by pipetting, and incubated on ice for 30 min. The reactions were quenched with 50 μl of 500 mM EDTA (pH 8.0), thus resulting in 50 to 200–base pair (bp) fragments. The digested lysates were placed in a 4°C centrifuge and spun for 10 min at top speed. To reduce potential noise, we precleared the lysates by mixing them with the beads that will be used to pull down protein-antibody complexes. The lysate (600 μl) was mixed 1:1 with 2× IP buffer [200 mM tris (pH 8.0), 600 mM NaCl, 4% (v/v) Triton X-100, 2× protease inhibitors] and 0.1 mg/ml molecular biology grade bovine serum albumin [BSA; New England Biolabs (NEB)]. We prepared NEB protein G beads (50 μl per sample) by washing them with 1 ml of 1× IP buffer without protease inhibitors but including 0.1 mg/ml molecular–biology-grade BSA and resuspending them in a final volume of 50 μl per sample. Washed beads were incubated with lysates at 4°C with rocking for 2 hours. Using a magnetic stand, beads were removed, and precleared lysates were placed into fresh tubes.

As an input control, 50 μl of precleared lysates were mixed with 450 μl of ChIP Elution Buffer [50 mM tris (pH 8.0), 10 mM EDTA, and 1% (w/v) SDS] and placed at 65°C for no more than 16 hours for cross-link reversal. DNA extraction will be described below. To the remainder of the lysate/IP buffer mixture, we added 5 μl of mCherry antibody (mCherry monoclonal antibody; Thermo Fisher Scientific, catalog no. M11217) and incubated the samples at 4°C overnight on a tube rocker. The next morning, we prepared a new batch of NEB protein G beads (50 μl per sample), distributed into antibody and lysate mixtures, and incubated at 4°C with rocking for 2 hours. The mixtures were then washed in the following series with 1-ml washes for each buffer and mixing by inversion. After each inversion, the tubes were placed on a magnetic stand to remove wash, and new wash was added. The following wash buffers were used in series: 1× Wash buffer A [100 mM tris (pH 8.0), 250 mM LiCl, 2% (v/v) Triton X-100, and 1 mM EDTA]; 1× Wash buffer B [100 mM tris (pH 8.0); 500 mM NaCl, 1% (v/v) Triton X-100, 0.1% (w/v) sodium deoxycholate, and 1 mM EDTA]; 1× Wash buffer C [10 mM tris (pH 8.0), 500 mM NaCl, 1% (v/v) Triton X-100, and 1 mM EDTA]; and 1× TE [10 mM tris (pH 8.0) and 1 mM EDTA]. After the last wash, beads were resuspended in 500 μl of ChIP Elution Buffer (recipe above) and incubated at 65°C for 30 min with vortexing every 5 to 10 min. The tubes were then placed on a magnetic stand; the supernatant was placed in a fresh tube, placed at 65°C for no more than 16 hours for cross-link reversal, and processed for DNA extraction as noted below. As a control, mCherry ChIP was performed on MG1655 strains lacking any tag, and the resulting signal from nonspecific binding was subtracted from analysis.

### DNA extraction after cross-link reversal

This procedure is the same as described in (*22*). Following incubation at 65°C, tubes were cooled and 100 μg of RNase A (Thermo Fisher Scientific) was added. The samples were incubated for 2 hours at 37°C. Then, 200 μg of proteinase K (Fermentas) was added, and the samples were incubated an additional 2 hours at 65°C. Phenol-chloroform extraction and ethanol precipitation were performed. During the ethanol precipitation, Glycoblue (Ambion) was used as a coprecipitant, NaCl was the precipitating salt, and washes were performed with ice-cold 95% (v/v) ethanol. The pellets were resuspended in 1× TEe [low-EDTA tris: 10 mM tris (pH 8 or 7.5) and 0.1 mM EDTA] and stored in DNA-Lobind tubes at −20°C.

### Preparation of next-generation sequencing libraries

Extracted DNA from the mCherry ChIP was prepared for sequencing using the NEBNext Ultra II Library Prep Kit (NEB) following the manufacturer’s instructions, with minor modifications: To purify complementary DNA, the Oligo Clean & Concentrator kit was used (Zymo). After the ligation of adapters, the DNA Clean & Concentrator-5 kit was used (Zymo). Dual-index primers for NEB were used in the sample preparation, and the libraries were sequences on an Illumina NextSeq. ChIP-seq data were preprocessed and quantified using the ChIP-seq portion of our IPOD-HR analysis software (https://github.com/freddolino-lab/ipod_v1_2020) to obtain ChIP versus input log_2_ ratios for each condition (merged across replicates). From there, we converted the log_2_ ratios to robust *z* scores and subtracted the observed robust *z* scores for samples obtained using untagged Hfq (which serve as a negative control) from those for samples with Hfq-mCherry, with values from the negative control clamped to be no lower than zero to avoid adding apparent occupancy. The resulting background-subtracted robust *z* scores were clamped to a minimum of zero (to consider only positive occupancy) and then used in subsequent analysis.

### RNA isolation and sequencing preparation

To isolate RNA, frozen pellets (described in previous section) were thawed on ice, resuspended in 100 μl of 1× TE, and treated with 177 kU of Ready-Lyse lysozyme solution (Lucigen) and 0.2 mg of proteinase K (Thermo Fisher Scientific). The mixture was incubated for 10 min at room temperature and vortexed every 2 min. The RNA was then purified using the RNA Clean & Concentrator-5 kit (Zymo) and treated with 5 U of Baseline-ZERO DNase (Epicentre) in the presence of an RNAse inhibitor (NEB) for 30 min at 37°C. The RNA purification was repeated using the RNA Clean & Concentrator-5 kit, and the resulting RNA was flash-frozen in a dry ice-ethanol bath and stored at −80°C. Ribosomal RNA (rRNA) depletion was performed on the stored RNA using the bacterial rRNA depletion kit following the manufacturer’s instructions (NEB), using the RNA Clean & Concentrator-5 kit instead of bead purification. The purified, rRNA-depleted RNA was then put through the NEBNext Ultra Directional RNA Library Prep Kit for Illumina following the manufacturer’s instructions (NEB) for rRNA-depleted RNA. We used random primers and considered the samples to be “intact” for the protocol specifications. Minor modifications to the protocol were the same as stated in the above next-generation sequencing library preparation. RNA-seq reads were aligned to the genome and quantified using kallisto 0.43 (*49*) and analyzed for differential expression using sleuth 0.30 (*50*).

### Data visualization and analysis tools

The following programs and websites were used for high-throughput data analysis and visualization: Numpy (*51*), R version 3.6.3 (www.rstudio.com/), tidyverse (www.tidyverse.org/), and ggplot2 (*52*).

### Hfq-PAmCherry Western blot

MG1655 hfq-PAmCherry and MG1655 Δ*ppk*-hfq-PAmCherry were grown to an OD_600_ of ~0.2 in MOPS-G. Before and 0.5, 1, 2, and 3 hours after treatment with cisplatin (10 μg/ml), 1 ml of each culture was removed, spun down, washed, resuspended in SDS–polyacrylamide gel electrophoresis loading buffer, and lysed by incubation at 90°C for 10 min. Proteins were then subjected to electrophoresis in a 4 to 15% SDS–polyacrylamide gel at 175V for 40 min. The gel was transferred onto an Immuno-Blot polyvinylidene difluoride membrane (Bio-Rad) at 25 V for 30 min. The membrane was blocked overnight with 5% (w/v) milk in TBST [10 mM tris-HCl (pH 8), 150 mM NaCl, and 0.05% (v/v) Tween 20) and incubated for ≥1 hour with primary anti-mcherry antibodies (1:10,000) (Thermo Fisher Scientific, catalog no. M11217) in blocking solution. Membranes were then rinsed with TBST five times and incubated for ≤1 hour with a 1:5000 dilution of anti-rat secondary antibody horseradish peroxidase (Thermo Fisher Scientific, no. 31470) in blocking solution. The membrane was rinsed with TBST three times and once with TBS and incubated with 500 μl of Western Lightning Plus-ECL chemiluminescence reagent (PerkinElmer). The membrane was imaged with Amersham Hyperfilm ECL (GE Healthcare). The hfq (host factor for RNA phage Q beta)-mcherry signal was normalized to total protein visualized by Coomassie staining of a 4 to 15% SDS–polyacrylamide gel run in parallel. The image quantification was performed using ImageJ. The experiments were conducted with three biological replicates.

### Hfq and Hfq-72 purification

*E. coli* BL21 (DE3) carrying pET-21a-*hfq*, pET-21a-*hfqS65C*, or pET-21a-*hfq-72* mutant were cultivated in LB and ampicillin at 37°C until an OD_600_ of 0.5 was reached. Protein expression was induced with 0.1 mM isopropyl-β-d-thiogalactopyranoside. After 16 hours of growth at 22°C, the cells were pelleted by centrifugation (5000*g*, 20 min, 4°C) and resuspended in lysis buffer [25 mM tris, 300 mM NaCl, DNase I (5 mg/ml), 5 μl of Benzonase nuclease (Merck), 100 μM MgCl_2_, and cOmplete (Roche) (pH 7.5)] and lysed by a 3-min sonication at 4°C. The lysates were incubated at room temperature for 30 min to ensure DNA degradation, transferred into boiling water for 20 min to precipitate most bacterial proteins, and transferred back to room temperature water for 15 min. The lysate was spun at 30,000*g*, 30 min, 4°C, and the supernatant was loaded onto two connected 5-ml HisTrap columns (Sigma-Aldrich). The columns were washed with 20 ml of buffer A [25 mM tris, 300 mM NaCl (pH 7.5)], 60 ml of 6 M guanidinium hydrochloride in buffer A, a 120-ml gradient from 6 to 0 M guanidinium hydrochloride in buffer A, followed by 60 ml of buffer A. Hfq was eluted with a 70-ml gradient from 15 to 300 mM imidazole in buffer A. The fractions containing highly pure Hfq were pooled and incubated for 1 hour with 100 μM MgCl_2_ and 4 μl of Benzonase before overnight dialysis against buffer A. After a 1:1 dilution of the sample into 25 mM tris (pH 7.5), the sample was loaded onto a 5-ml HiTrap SP HP column (Sigma-Aldrich) and washed with 5% buffer B [25 mM tris and 100 mM NaCl (pH 7.5)]. Hfq was eluted with a gradient from 6 to 40% buffer C [25 mM tris and 1.0 M NaCl (pH 7.5)]. Hfq-72 eluted in the flow-through. The purified samples were pooled, dialyzed against 25 mM tris and 300 mM NaCl (pH 7.5), and stored at −80°C. The OD_280/260_ ratio of Hfq was ~1.7.

### Sedimentation velocity analytical ultracentrifugation

For the analytical ultracentrifugation (AUC), 420 μl of sample containing 25 μM Hfq ± 250 μM polyP_60_ or polyP_300_ (in Pi units) in 20 mM Hepes, 100 mM NaCl (pH 8.0) was loaded into epon-charcoal two-channel centerpieces with 1.2-cm path length in an An60Ti rotor in a Beckman Optima Xl-I analytical ultracentrifuge. Measurements were completed in intensity mode at 32,000 rpm for the Hfq-polyP samples and at 48,000 rpm for Hfq alone. Absorbance at 280 nm was monitored. All sedimentation velocity–AUC data were analyzed using UltraScan 4 software (version 4.0), and fitting procedures were completed on XSEDE clusters at the Texas Advanced Computing Center (Lonestar, Stampede, Jetstrean) through the UltraScan Science Gateway (www.xsede.org/web/guest/gateways-listing) (*53*). The partial specific volume (vbar) of Hfq was estimated within UltraScan III on the basis of the protein sequence (*54*). Raw intensity data were converted to pseudo-absorbance by using the intensity of the air above the meniscus as a reference and edited. Next, two-dimensional sedimentation spectrum analysis (2DSA) was performed to subtract time-invariant noise, and the meniscus was fit using 10 points in a 0.05-cm range (*55*). First arrays with a broad S range were fitted to account for possible large oligomeric states. Final arrays were fit using a broad S range from 1 to 50 for the complex and 1 to 10 for Hfq, an *f*/*f*_0_ range of 1 to 4 with 64 grid points for each, 10 uniform grid repetitions, and 400 simulation points. 2DSA was then repeated at the determined meniscus to fit radially invariant and time-invariant noise together using 10 iterations. The 2DSA solution was refined by a genetic algorithm, which uses an evolutionary-based approach using random crossover, mutations, and deletion operations to alter the solute characteristics of the 2DSA solutes to eliminate false-positive solutions.

### PolyP, DNA, and Hfq labeling

Different polyP chain lengths (i.e., polyP_16_, polyP_60_, polyP_130_, polyP_300_, and polyP_700_) were provided by T. Shiba (RegeneTiss, Japan). If not otherwise stated, all buffers and chemicals were from Sigma-Aldrich. PolyP_300_ was labeled with AF647 as previously described (*56*), applying some minor modifications. Briefly, 37.5 mM polyP_300_ (in P_i_ units) was incubated with 1 mg of AF647 cadaverine (Life Technologies) and 200 mM 1-ethyl-3-(3-dimethylaminopropyl) carbodiimide (Invitrogen) in 20 mM MOPS and 100 mM NaCl (pH 8.0) at 45°C for 1 hour. The reaction was stopped on ice. Labeled AF647-polyP_300_ was separated from free dye using a PD-10 column (GE Healthcare) equilibrated with the same buffer. The collected fractions were pooled and cleaned up in 50-μl aliquots using a Zeba desalting column (Qiagen). The concentration of polyP was determined in 100-μl samples, containing 20 mM MOPS, 100 mM NaCl (pH 8.0), and 1 μg of DAPI, using an excitation wavelength of 415 and an emission wavelength of 550 nm (*57*). A polyP standard was used. The analysis of the AF647 fluorescence in the same samples revealed a labeling efficiency between 0.1 to 0.2% polyP. Three different DNA sequences were generated by polymerase chain reaction (PCR), double-stranded DNA (dsDNA) covering a polyP-dependent in vivo binding site encompassing the *ybck* gene, a 180-bp-long dsDNA covering a *ppk*-dependent in vivo Hfq binding site, and a 183-bp-long dsDNA covering an in vivo nonbinding site of Hfq. For labeling, FAM-labeled forward and reverse primers were used for the *ybck*-DNA and the *ppk*-independent DNA, while Cy5-labeled forward and reverse primers were used for the nonbinding sequence. HfqS65C was labeled with Cy3 Maleimide Mono-Reactive Dye (Amersham) following the manufacturer’s protocol. Briefly, ~1 mg of purified HfqS65C was reduced with 180 μg of tris-(2-carboxyethyl)phosphine hydrochloride in 50 mM K_2_HPO_4_ (pH 7.5) buffer containing 100 mM NaCl. One pack of dye was dissolved in 50 μl of anhydrous dimethyl sulfoxide, and the entire dye solution was added to reduced HfqS65C yielding a final reaction volume of 1 ml. The reaction was incubated for 2 hours at room temperature and then left overnight at 4°C. The labeled protein was separated from the excess dye by passing it through a PD-10 desalting column packed with Sephadex G-25 resin equilibrated with the same buffer as the labeling reaction. The labeled protein was eluted in the same buffer and concentrated using an Amicon Ultra centrifugal filter with 3-kDa MW cutoff. The concentration of labeled protein and dye/protein ratio were calculated using a molar extinction coefficient of 150,000 M^−1^ cm^−1^ at 552 nm for Cy3 and a correction factor of 0.08 for dye absorbance at 280 nm. The labeling efficiency was ~23%, similar to previous work with this Hfq mutant (*31*).

### RNA generation

PCR was performed on MG1655 genomic DNA using essD_T7_F and essD_T7_R primers that included a T7 RNA Polymerase promoter for transcription. The HiScribeTM T7 High Yield RNA Synthesis Kit (NEB, E2040S) was used to generate RNA following the manufacturer’s instructions. RNA was the purified as described above in the “RNA isolation and sequencing preparation” section, beginning from the use of the first RNA Clean & Concentrator-5 kit.

### Native gel electrophoresis and electrophoretic mobility shift assay

To determine the interaction of polyP and Hfq on tris-borate EDTA (TBE) 4 to 20% native gels, 25 μM Hfq protein was incubated with the indicated concentrations of polyP in 20 mM Hepes and 100 mM NaCl (pH 8.0). However, similar results were obtained in 25 mM tris, 200 mM NaCl (pH 7.5) or 25 mM tris, 30 mM NaCl. For electrophoretic mobility shift assay assays, the indicated concentrations of the respective DNA or RNA fragments were incubated in the absence or presence of the indicated concentrations of Hfq or Hfq-72 and polyP_300_ in 25 mM tris and 200 mM NaCl (pH 7.5). After adding glycerol [10% (v/v) final concentration] and loading dye (NEB), samples were run on a 4 to 20% TBE gel (Invitrogen) at 4°C (70 V, 16 hours or 100 V, 4 hours) using TBE (pH 8.3) buffer. DNA was stained with SybrSafe (Invitrogen), while polyP was stained with DAPI (*57*) unless FAM-labeled DNA and/or AF647-labeled polyP_300_ was used. In that case, the gels were analyzed using a fluorescence imager. Hfq was visualized using Coomassie stain (*58*).

### Preparation of LLPS for microscopic analysis and FRAP studies

Five microliters of purified Hfq (0.5 to 4 mM in 25 mM tris and 300 mM NaCl (pH 7.5) supplemented with 8 to 33 μM Cy3-labeled Hfq] was diluted 1:10 into ddH_2_O in the absence or presence of 100 nM FAM-labeled DNA and/or 100 μM AF647-polyP_300_ as indicated in the figure legends. Five microliters of Hfq-72 (250 μM in 25 mM tris and 300 mM NaCl (pH 7.5) supplemented with 8 μM Cy3-labeled Hfq-S65C) was diluted 1:10 into ddH_2_O. For C_sat_ measurements, Cy3-labeled Hfq was supplemented 1:75 into nonlabeled Hfq. Hfq was serially diluted into 25 mM tris and 200 mM NaCl (pH 7.5). Five microliters of each sample was diluted into ddH_2_O in the absence or presence of equimolar amounts of polyP. The samples were analyzed in 16-well CultureWell with glass bottoms (Grace BioLabs), passivated by overnight incubation with 5% (w/v) pluronic acid (Thermo Fisher Scientific), and washed thoroughly with ddH_2_O before use. Time-lapse movies and FRAP measurements were performed after 40-min incubation. All fluorescence and differential interference contrast (DIC) imaging were performed using a Nikon Ti2-E motorized inverted microscope controlled by NIS Elements software with a SOLA 365 LED light source, a 100× objective lens (Oil CFI60 Plan Apochromat Lambda Series for DIC), and a Photometrics Prime 95B Back-illuminated sCMOS camera. FAM-labeled DNA was imaged using a “GFP” filter set [excitation, 470/40 nm (450 to 490 nm); emission, 525/50 nm (500 to 550 nm); dichroic mirror, 495 nm]. mCherry-Hfq was imaged using a “Texas Red” filter set [excitation, 560/40 nm (540 to 580 nm); emission, 630/70 nm (593 to 668 nm); dichroic mirror. 585 nm]. AF647-polyP was imaged using a “CY5” filter set [excitation, 620/60 nm (590 to 650 nm); emission, 700/75 nm (663 to 738 nm); dichroic mirror, 660 nm]. Bleaching was conducted with a 405-nm laser at 40% power (20 mW) with a 200-μs dwell time. Time-lapse images and videos were taken at 10% light intensity with 500-ms exposures. The time lapse was set up to take a single prebleach image, followed by bleaching and image acquisition every 5 s for 5 min. Fifteen different regions of interest (ROIs) were chosen per time point, with five droplets each being completely bleached (“full-bleach”), half-bleached, or center-bleached. At least one ROI in the nonbleached area was selected and analyzed. One ROI in an adjacent, nonbleached droplet was selected and served as a control. Image analysis was performed using ImageJ. Data were exported, further tabulated, graphed, and analyzed using GraphPad Prism 9.0.1 (GraphPad Software, San Diego, California, USA; www.graphpad.com). Intensities were normalized using an unbleached droplet to account for any full–field of view photobleaching. The data points immediately following the bleach step were fit to a one-phase association least squares fit to obtain the indicated half-times (*n* ≥ 3 droplets per condition).

### Preparation of LLPS for turbidity measurements

To analyze LLPS formation using turbidity measurements, Hfq or Hfq-72 in 25 mM tris and 300 mM NaCl (pH 7.5) was diluted 1:10 into 25 mM tris buffer (pH 7.5) supplemented with the indicated concentrations of NaCl, DNA, polyP, and/or 10% (w/v) Ficoll 70 (Sigma-Aldrich) at room temperature (details are given in the figure legends). Turbidity measurements were conducted in 96-well plates at 350 nm using an Infinite1000 (Tecan). All experiments were conducted at least in triplicate.

### Single-molecule fluorescence microscopy

Single-molecule tracking of Hfq-PAmCherry was performed on a wide-field Olympus IX71 inverted microscope with 1.40–numerical aperture 100× oil immersion objective and detected on a 512 × 512 pixel Photometrics Evolve electron-multiplying CCD at a rate of 50 Hz. WT and **∆***ppk* Hfq-PAmCherry strains were cultivated in MOPS-G medium to reach OD 0.5 for imaging. Cells were imaged after immobilization on 2% (w/v) agarose pads. The Hfq-PAmCherry was photoactivated with 100-ms pulses of a 405-nm laser (Coherent Cube, 405-100; 0.4 W/cm^2^) and then imaged under excitation by a 561-nm laser (Coherent-Sapphire, 561-50; 70 W/cm^2^). The recorded movies were analyzed to localize and track single molecules with the SMALL-LABS algorithm (*59*) to acquire single-molecule trajectory datasets. The Spot-On algorithm (*24*) was applied to fit the probability density function to a three-state model to quantify the single-molecule dynamics of Hfq. For both WT and **∆***ppk* strains, >50,000 single-molecule steps from >7000 trajectories in >150 cells were used for Spot-On fitting.

## Supplementary Materials

### This PDF file includes:

Figs. S1 to S4 Table S1 Legends for movies S1 to S7 Legends for data S1 and S2 References

## Notes

View/request a protocol for this paper from *Bio-protocol*.

## References

[1] M. R. Brown, A. Kornberg, Inorganic polyphosphate in the origin and survival of species. Proc. Natl. Acad. Sci. U.S.A. 101, 16085–16087 (2004). 15520374 10.1073/pnas.0406909101 PMC528972

[2] L. Xie, A. Rajpurkar, E. Quarles, N. Taube, A. S. Rai, J. Erba, B. Sliwinski, M. Markowitz, U. Jakob, D. Knoefler, Accumulation of nucleolar inorganic polyphosphate is a cellular response to cisplatin-induced apoptosis. Front. Oncol. 9, 1410 (2019). 31921667 10.3389/fonc.2019.01410 PMC6920253

[3] M. J. Gray, W. Y. Wholey, N. O. Wagner, C. M. Cremers, A. Mueller-Schickert, N. T. Hock, A. G. Krieger, E. M. Smith, R. A. Bender, J. C. A. Bardwell, U. Jakob, Polyphosphate is a primordial chaperone. Mol. Cell 53, 689–699 (2014). 24560923 10.1016/j.molcel.2014.01.012 PMC3996911

[4] F. Beaufay, E. Quarles, A. Franz, O. Katamanin, W.-Y. Wholey, U. Jakob, Polyphosphate functions in vivo as an iron chelator and fenton reaction inhibitor. MBio 11, 4 (2020). 10.1128/mBio.01017-20 PMC7387796 32723918

[5] A. Du Toit, Phage induction in different contexts. Nat. Rev. Microbiol. 17, 126–127 (2019). 10.1038/s41579-019-0150-4 30683886

[6] G. Pósfai, G. Plunkett III, T. Fehér, D. Frisch, G. M. Keil, K. Umenhoffer, V. Kolisnychenko, B. Stahl, S. S. Sharma, M. de Arruda, V. Burland, S. W. Harcum, F. R. Blattner, Emergent properties of reduced-genome *Escherichia coli*. Science 312, 1044–1046 (2006). 16645050 10.1126/science.1126439

[7] R. P. Rastogi, Richa, A. Kumar, M. B. Tyagi, R. P. Sinha, Molecular mechanisms of ultraviolet radiation-induced DNA damage and repair. J. Nucleic Acids 2010, 592980 (2010). 21209706 10.4061/2010/592980 PMC3010660

[8] T. Feher, B. Cseh, K. Umenhoffer, I. Karcagi, G. Posfai, Characterization of cycA mutants of *Escherichia coli*. An assay for measuring in vivo mutation rates. Mutat. Res. 595, 184–190 (2006). 16376388 10.1016/j.mrfmmm.2005.11.004

[9] D. J. Jin, C. A. Gross, Mapping and sequencing of mutations in the *Escherichia coli* rpoB gene that lead to rifampicin resistance. J. Mol. Biol. 202, 45–58 (1988). 3050121 10.1016/0022-2836(88)90517-7

[10] N. N. Rao, M. R. Gomez-Garcia, A. Kornberg, Inorganic polyphosphate: Essential for growth and survival. Annu. Rev. Biochem. 78, 605–647 (2009). 19344251 10.1146/annurev.biochem.77.083007.093039

[11] K. Murata, S. Hagiwara, Y. Kimori, Y. Kaneko, Ultrastructure of compacted DNA in cyanobacteria by high-voltage cryo-electron tomography. Sci. Rep. 6, 34934 (2016). 27731339 10.1038/srep34934 PMC5059737

[12] R. T. Dame, The role of nucleoid-associated proteins in the organization and compaction of bacterial chromatin. Mol. Microbiol. 56, 858–870 (2005). 15853876 10.1111/j.1365-2958.2005.04598.x

[13] J. McQuail, A. Switzer, L. Burchell, S. Wigneshweraraj, The RNA-binding protein Hfq assembles into foci-like structures in nitrogen starved *Escherichia coli*. J. Biol. Chem. 295, 12355–12367 (2020). 32532816 10.1074/jbc.RA120.014107 PMC7458820

[14] I. Moll, D. Leitsch, T. Steinhauser, U. Blasi, RNA chaperone activity of the Sm-like Hfq protein. EMBO Rep. 4, 284–289 (2003). 12634847 10.1038/sj.embor.embor772 PMC1315900

[15] K. M. Wassarman, F. Repoila, C. Rosenow, G. Storz, S. Gottesman, Identification of novel small RNAs using comparative genomics and microarrays. Genes Dev. 15, 1637–1651 (2001). 11445539 10.1101/gad.901001 PMC312727

[16] J. Orans, A. R. Kovach, K. E. Hoff, N. M. Horstmann, R. G. Brennan, Crystal structure of an *Escherichia coli* Hfq Core (residues 2-69)-DNA complex reveals multifunctional nucleic acid binding sites. Nucleic Acids Res. 48, 3987–3997 (2020). 32133526 10.1093/nar/gkaa149 PMC7144919

[17] A. Takada, M. Wachi, A. Kaidow, M. Takamura, K. Nagai, DNA binding properties of the hfq gene product of *Escherichia coli*. Biochem. Biophys. Res. Commun. 236, 576–579 (1997). 9245691 10.1006/bbrc.1997.7013

[18] M. Kajitani, A. Kato, A. Wada, Y. Inokuchi, A. Ishihama, Regulation of the *Escherichia coli* hfq gene encoding the host factor for phage Q beta. J. Bacteriol. 176, 531–534 (1994). 8288550 10.1128/jb.176.2.531-534.1994 PMC205081

[19] G. W. Li, D. Burkhardt, C. Gross, J. S. Weissman, Quantifying absolute protein synthesis rates reveals principles underlying allocation of cellular resources. Cell 157, 624–635 (2014). 24766808 10.1016/j.cell.2014.02.033 PMC4006352

[20] A. Zhang, D. J. Schu, B. C. Tjaden, G. Storz, S. Gottesman, Mutations in interaction surfaces differentially impact E. coli Hfq association with small RNAs and their mRNA targets. J. Mol. Biol. 425, 3678–3697 (2013). 23318956 10.1016/j.jmb.2013.01.006 PMC3640674

[21] T. Vora, A. K. Hottes, S. Tavazoie, Protein occupancy landscape of a bacterial genome. Mol. Cell 35, 247–253 (2009). 19647521 10.1016/j.molcel.2009.06.035 PMC2763621

[22] P. L. Freddolino, H. M. Amemiya, T. J. Goss, S. Tavazoie, Dynamic landscape of protein occupancy across the *Escherichia coli* chromosome. PLoS Biol. 19, e3001306 (2021). 34170902 10.1371/journal.pbio.3001306 PMC8282354

[23] H. M. Amemiya, T. J. Goss, T. M. Nye, R. Hurto, L. A. Simmons, P. L. Freddolino, Distinct heterochromatin-like domains promote transcriptional memory and silence parasitic genetic elements in bacteria. *EMBO J.*, in press. 10.15252/embj.2021108708 PMC8804932 34961960

[24] C. Sauter, J. Basquin, D. Suck, Sm-like proteins in Eubacteria: The crystal structure of the Hfq protein from *Escherichia coli*. Nucleic Acids Res. 31, 4091–4098 (2003). 12853626 10.1093/nar/gkg480 PMC167641

[25] S. Melamed, A. Peer, R. Faigenbaum-Romm, Y. E. Gatt, N. Reiss, A. Bar, Y. Altuvia, L. Argaman, H. Margalit, Global mapping of small RNA-target interactions in bacteria. Mol. Cell 63, 884–897 (2016). 27588604 10.1016/j.molcel.2016.07.026 PMC5145812

[26] A. E. Posey, A. S. Holehouse, R. V. Pappu, Phase separation of intrinsically disordered proteins. Methods Enzymol. 611, 1–30 (2018). 30471685 10.1016/bs.mie.2018.09.035

[27] F. Erdel, K. Rippe, Formation of chromatin subcompartments by phase separation. Biophys. J. 114, 2262–2270 (2018). 29628210 10.1016/j.bpj.2018.03.011 PMC6129460

[28] T. Ali Azam, A. Iwata, A. Nishimura, S. Ueda, A. Ishihama, Growth phase-dependent variation in protein composition of the *Escherichia coli* nucleoid. J. Bacteriol. 181, 6361–6370 (1999). 10515926 10.1128/jb.181.20.6361-6370.1999 PMC103771

[29] T. B. Updegrove, J. J. Correia, R. Galletto, W. Bujalowski, R. M. Wartell, E. coli DNA associated with isolated Hfq interacts with Hfq’s distal surface and C-terminal domain. Biochim. Biophys. Acta 1799, 588–596 (2010). 20619373 10.1016/j.bbagrm.2010.06.007 PMC3072145

[30] T. B. Updegrove, A. Zhang, G. Storz, Hfq: The flexible RNA matchmaker. Curr. Opin. Microbiol. 30, 133–138 (2016). 26907610 10.1016/j.mib.2016.02.003 PMC4821791

[31] S. Panja, S. A. Woodson, Fluorescence reporters for Hfq oligomerization and RNA annealing. Methods Mol. Biol. 1259, 369–383 (2015). 25579597 10.1007/978-1-4939-2214-7_22 PMC4340055

[32] H. H. Tuson, J. S. Biteen, Unveiling the inner workings of live bacteria using super-resolution microscopy. Anal. Chem. 87, 42–63 (2015). 25380480 10.1021/ac5041346

[33] A. S. Hansen, M. Woringer, J. B. Grimm, L. D. Lavis, R. Tjian, X. Darzacq, Robust model-based analysis of single-particle tracking experiments with Spot-On. eLife 7, e33125 (2018). 29300163 10.7554/eLife.33125 PMC5809147

[34] M. Yousuf, I. Iuliani, R. T. Veetil, A. S. N. Seshasayee, B. Sclavi, M. C. Lagomarsino, Early fate of exogenous promoters in E. coli. Nucleic Acids Res. 48, 2348–2356 (2020). 31960057 10.1093/nar/gkz1196 PMC7049719

[35] W. W. Navarre, S. Porwollik, Y. Wang, M. McClelland, H. Rosen, S. J. Libby, F. C. Fang, Selective silencing of foreign DNA with low GC content by the H-NS protein in Salmonella. Science 313, 236–238 (2006). 16763111 10.1126/science.1128794

[36] B. A. Shen, R. Landick, Transcription of bacterial chromatin. J. Mol. Biol. 431, 4040–4066 (2019). 31153903 10.1016/j.jmb.2019.05.041 PMC7248592

[37] K. Singh, J. N. Milstein, W. W. Navarre, Xenogeneic silencing and its impact on bacterial genomes. Annu. Rev. Microbiol. 70, 199–213 (2016). 27359215 10.1146/annurev-micro-102215-095301

[38] T. A. Azam, A. Ishihama, Twelve species of the nucleoid-associated protein from *Escherichia coli*. Sequence recognition specificity and DNA binding affinity. J. Biol. Chem. 274, 33105–33113 (1999). 10551881 10.1074/jbc.274.46.33105

[39] A. Malabirade, K. Jiang, K. Kubiak, A. Diaz-Mendoza, F. Liu, J. A. van Kan, J. F. Berret, V. Arluison, J. R. C. van der Maarel, Compaction and condensation of DNA mediated by the C-terminal domain of Hfq. Nucleic Acids Res. 45, 7299–7308 (2017). 28521053 10.1093/nar/gkx431 PMC5499573

[40] E. Fortas, F. Piccirilli, A. Malabirade, V. Militello, S. Trépout, S. Marco, A. Taghbalout, V. Arluison, New insight into the structure and function of Hfq C-terminus. Biosci. Rep. 35, e00190 (2015). 25772301 10.1042/BSR20140128 PMC4413018

[41] A. G. Larson, D. Elnatan, M. M. Keenen, M. J. Trnka, J. B. Johnston, A. L. Burlingame, D. A. Agard, S. Redding, G. J. Narlikar, Liquid droplet formation by HP1α suggests a role for phase separation in heterochromatin. Nature 547, 236–240 (2017). 28636604 10.1038/nature22822 PMC5606208

[42] A. R. Strom, A. V. Emelyanov, M. Mir, D. V. Fyodorov, X. Darzacq, G. H. Karpen, Phase separation drives heterochromatin domain formation. Nature 547, 241–245 (2017). 28636597 10.1038/nature22989 PMC6022742

[43] F. R. Blattner, G. Plunkett III, C. A. Bloch, N. T. Perna, V. Burland, M. Riley, J. Collado-Vides, J. D. Glasner, C. K. Rode, G. F. Mayhew, J. Gregor, N. W. Davis, H. A. Kirkpatrick, M. A. Goeden, D. J. Rose, B. Mau, Y. Shao, The complete genome sequence of *Escherichia coli* K-12. Science 277, 1453–1462 (1997). 9278503 10.1126/science.277.5331.1453

[44] K. A. Datsenko, B. L. Wanner, One-step inactivation of chromosomal genes in *Escherichia coli* K-12 using PCR products. Proc. Natl. Acad. Sci. U.S.A. 97, 6640–6645 (2000). 10829079 10.1073/pnas.120163297 PMC18686

[45] J. Salvatier, T. V. Wiecki, C. Fonnesbeck, Probabilistic programming in Python using PyMC3. Peer J. Comput. Sci. 2, e55 (2016).

[46] A. Ducret, E. M. Quardokus, Y. V. Brun, MicrobeJ, a tool for high throughput bacterial cell detection and quantitative analysis. Nat. Microbiol. 1, 16077 (2016). 27572972 10.1038/nmicrobiol.2016.77 PMC5010025

[47] E. Laskowska, J. Bohdanowicz, D. Kuczyńska-Wiśnik, E. Matuszewska, S. Kędzierska, A. Taylor, Aggregation of heat-shock-denatured, endogenous proteins and distribution of the IbpA/B and Fda marker-proteins in *Escherichia coli* WT and grpE280 cells. Microbiology 150, 247–259 (2004). 14702418 10.1099/mic.0.26470-0

[48] J. Miller, *A Short Course in Bacterial Genetics–A Laboratory Manual and Handbook for Escherichia coli and Related Bacteria* (Cold Spring Harbor Laboratory Press, 1992).

[49] N. L. Bray, H. Pimentel, P. Melsted, L. Pachter, Near-optimal probabilistic RNA-seq quantification. Nat. Biotechnol. 34, 525–527 (2016). 27043002 10.1038/nbt.3519

[50] H. Pimentel, N. L. Bray, S. Puente, P. Melsted, L. Pachter, Differential analysis of RNA-seq incorporating quantification uncertainty. Nat. Methods 14, 687–690 (2017). 28581496 10.1038/nmeth.4324

[51] C. R. Harris, K. J. Millman, S. J. van der Walt, R. Gommers, P. Virtanen, D. Cournapeau, E. Wieser, J. Taylor, S. Berg, N. J. Smith, R. Kern, M. Picus, S. Hoyer, M. H. van Kerkwijk, M. Brett, A. Haldane, J. F. del Río, M. Wiebe, P. Peterson, P. Gérard-Marchant, K. Sheppard, T. Reddy, W. Weckesser, H. Abbasi, C. Gohlke, T. E. Oliphant, Array programming with NumPy. Nature 585, 357–362 (2020). 32939066 10.1038/s41586-020-2649-2 PMC7759461

[52] H. Wickham, *ggplot2 - Elegant Graphics for Data Analysis* (Springer, 2009).

[53] B. Demeler, G. E. Gorbet, Analytical ultracentrifugation data analysis with UltraScan-III, in *Analytical Ultracentrifugation*, S. Uchiyama, F. Arisaka, W. F. Stafford, T. Laue, Eds. (Springer, 2016), pp. 119–143.

[54] B. Demeler, E. Brookes, L. Nagel-Steger, Analysis of heterogeneity in molecular weight and shape by analytical ultracentrifugation using parallel distributed computing. Methods Enzymol. 454, 87–113 (2009). 19216924 10.1016/S0076-6879(08)03804-4

[55] E. Brookes, W. Cao, B. Demeler, A two-dimensional spectrum analysis for sedimentation velocity experiments of mixtures with heterogeneity in molecular weight and shape. Eur. Biophys. J. 39, 405–414 (2010). 19247646 10.1007/s00249-009-0413-5 PMC12146834

[56] J. Lempart, E. Tse, J. A. Lauer, M. I. Ivanova, A. Sutter, N. Yoo, P. Huettemann, D. Southworth, U. Jakob, Mechanistic insights into the protective roles of polyphosphate against amyloid cytotoxicity. Life Sci. Alliance 2, e201900486 (2019). 31533964 10.26508/lsa.201900486 PMC6751573

[57] S. A. Smith, J. H. Morrissey, Sensitive fluorescence detection of polyphosphate in polyacrylamide gels using 4′,6-diamidino-2-phenylindol. Electrophoresis 28, 3461–3465 (2007). 17847128 10.1002/elps.200700041

[58] C. Wong, S. Sridhara, J. C. Bardwell, U. Jakob, Heating greatly speeds Coomassie blue staining and destaining. Biotechniques 28, 426–428, 430, 432 (2000). 10.2144/00283bm07 10723553

[59] B. P. Isaacoff, Y. Li, S. A. Lee, J. S. Biteen, SMALL-LABS: Measuring single-molecule intensity and position in obscuring backgrounds. Biophys. J. 116, 975–982 (2019). 30846363 10.1016/j.bpj.2019.02.006 PMC6428939

[60] L. M. Guzman, D. Belin, M. J. Carson, J. Beckwith, Tight regulation, modulation, and high-level expression by vectors containing the arabinose PBAD promoter. J. Bacteriol. 177, 4121–4130 (1995). 7608087 10.1128/jb.177.14.4121-4130.1995 PMC177145

