# Supplementary material for: Polyphosphate drives bacterial heterochromatin formation
Source: Sci Adv. 2021 Dec 22;7(52):eabk0233. doi: 10.1126/sciadv.abk0233 (PMC10954037; doi:10.1126/sciadv.abk0233)
Supplement: Supplementary file 1 — Figs. S1 to S4 Table S1 Legends for movies S1 to S7 Legends for data S1 and S2 References [file sciadv.abk0233_sm.pdf]

Science Advances
AAAS

Supplementary Materials for

Polyphosphate drives bacterial heterochromatin formation

Francois Beaufay, Haley M. Amemiya, Jian Guan, Joseph Basalla, Ben A. Meinen, Ziyuan Chen, Rishav Mitra, James C. A. Bardwell, Julie S. Biteen, Anthony G. Vecchiarelli, Lydia Freddolino*, Ursula Jakob*

*Corresponding author. Email: ujakob@umich.edu (U.J.); lydsf@umich.edu (L.F.)

Published 22 December 2021, *Sci. Adv.* **7**, eabk0233 (2021)
DOI: 10.1126/sciadv.abk0233

The PDF file includes:

Figs. S1 to S4
Table S1
Legends for movies S1 to S7
Legends for data S1 and S2
References

Other Supplementary Material for this manuscript includes the following:

Movies S1 to S7
Data S1 and S2

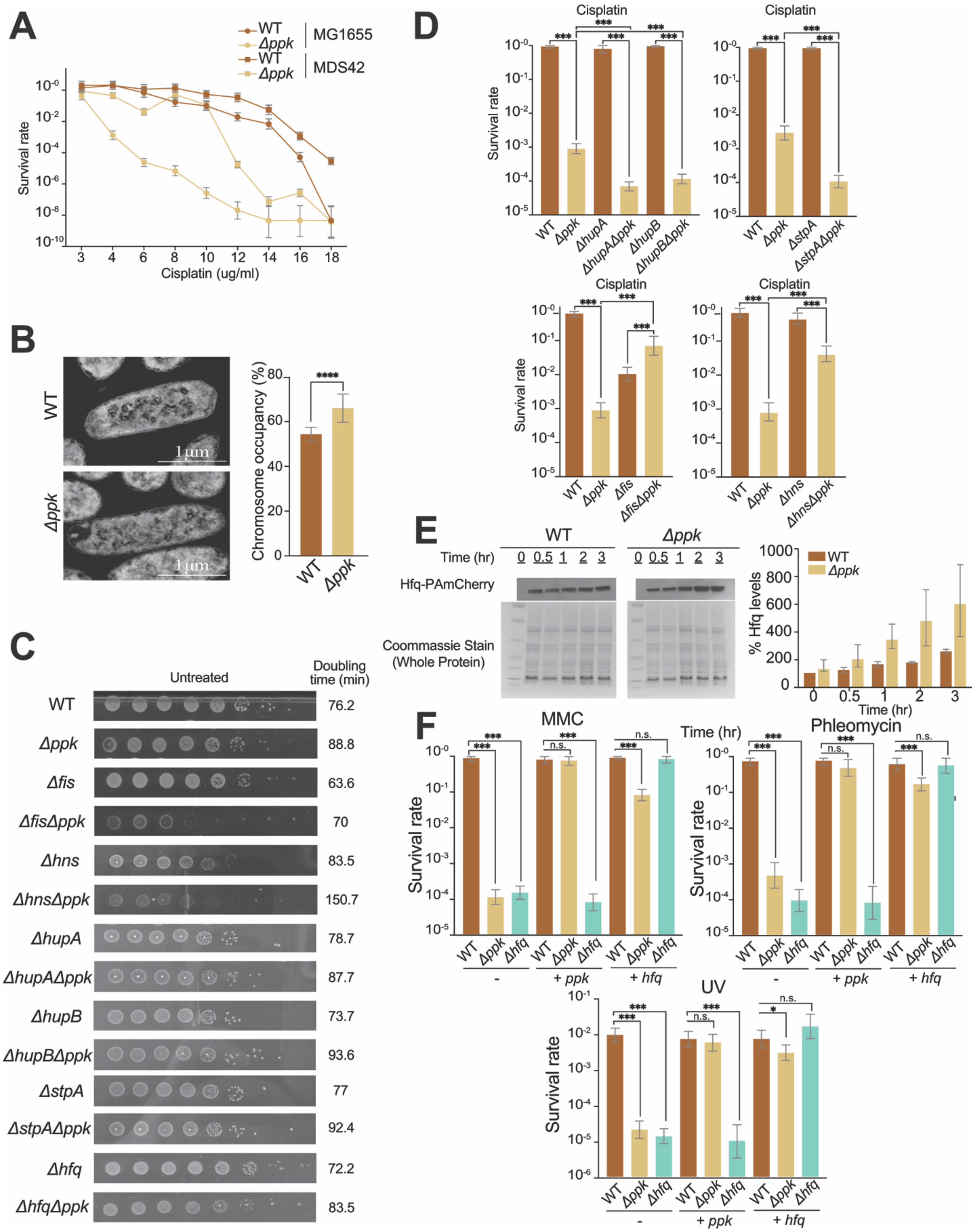

Fig. S1. *Ppk* promotes cells survival upon DNA damaging stress conditions
(A) Survival of MG1655 and MDS42 and their respective *ppk* deletion mutants upon exposure to increasing concentrations of cisplatin. (**B**) Left panel: thin section microscopy of MG1655 WT and *Δppk* cells; right panel: associated space occupied by the nucleoid in each mutant under non-stress conditions.

(**C**) 10X serial dilution of various NAP deletion strains in otherwise MG1655 wild-type or Δ*ppk* background, and their associate growth rates in MOPS-glucose media. (**D**) Survival of the indicated strains in an MG1655 background upon exposure to 4 μg/ml cisplatin. *** indicates a greater than 99.5% posterior probability that the indicated survival rates differ (using a Bayesian analysis of the survival data; see Methods for details). **E)** Abundance of Hfq-PA-mcherry in wild-type and *Δppk* cells before and at defined times after addition of 10 μg/ml cisplatin as determined by western blot analysis using antibodies against mCherry. The total protein loaded on the SDS-PAGE was used as loading control. The steady state level of Hfq at time point 0 in Hfq-PA-mcherry expressing strain MG1655 was set to 100%. **(F)** Survival of wild-type, *Δppk* and *Δhfq* cells in MG1655 complemented with an empty plasmid or a plasmid carrying either a copy of *ppk* or *hfq* upon exposure to 4 μg/ml cisplatin, 1 μg/ml mitomycin C (MMC), 0.25 μg/ml phleomycin or 25 J/m^2 UV. Survival rates were scored after 16-24 h growth at 37°C. Significance is indicated based on the fraction of the posterior density for the difference between a WT background and deletion mutants (bearing the same overexpression plasmid) that is in the observed direction: *, P>0.95; **, P>0.99; ***, P>0.995; ****, P>0.9995.

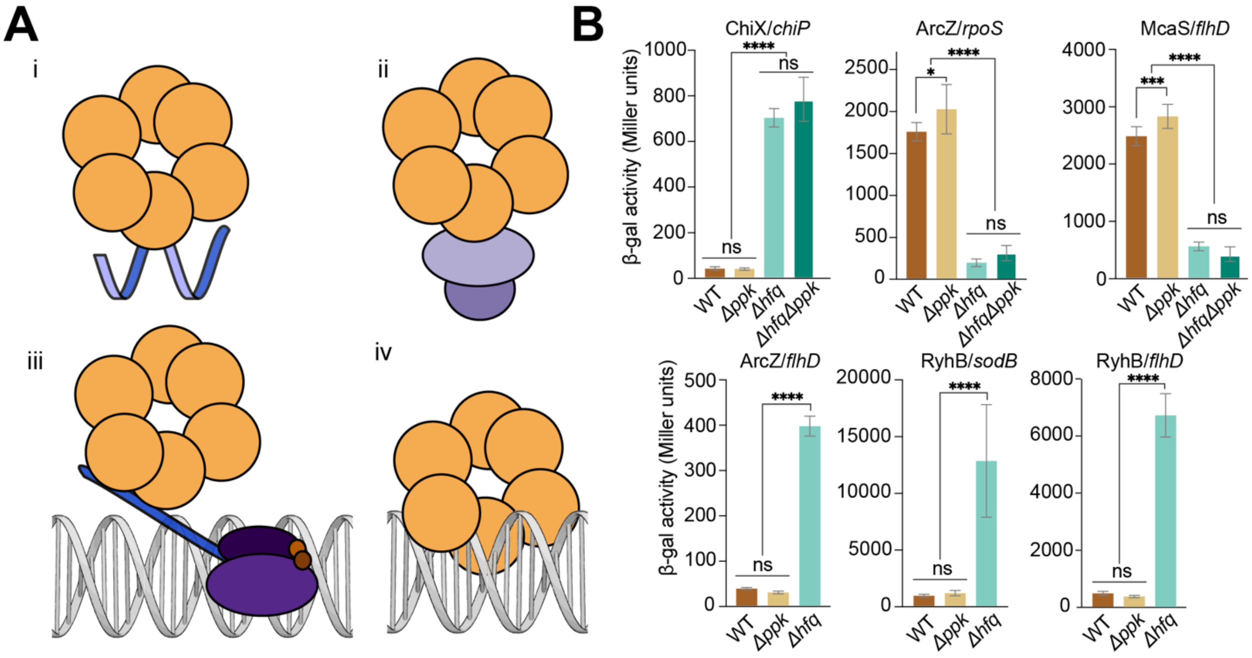

Fig. S2. Known functions of Hfq and effect of polyP on *in vivo* RNA chaperone activity of Hfq.
(A) Schematic of known mechanisms through which Hfq interacts with nucleic acids; (i) direct binding of RNA (multiple binding faces exist, which may lead to either small RNA-mediated or direct effects on the target); (ii) chaperone activity in ribosome biogenesis; (iii) indirect association with DNA through interactions with nascent transcripts; (iv) direct binding of DNA. Citations describing each role are given in the main text. (**B)** Activity of small RNAs on their specific targets in *Δppk* and *Δhfq* cells measured by β-galactosidase assays (n≥3; *, P>0.95; **, P>0.99; ***, P>0.995; ****, P>0.9995; ns, nonsignificant, tukey's multiple comparisons test).

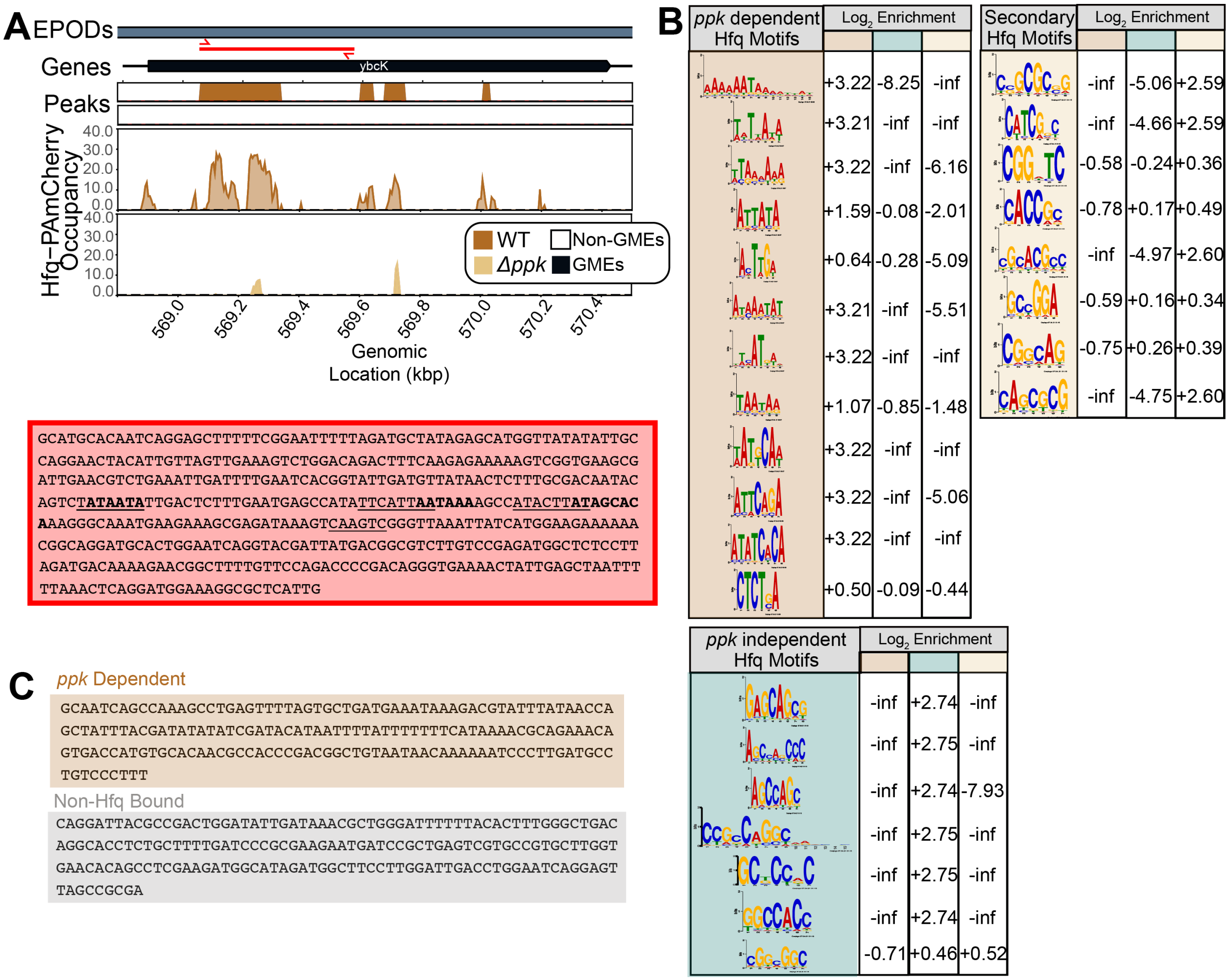

Fig. S3. Absence of *ppk* changes DNA binding specificity of Hfq
(A) Genomic region containing a *ppk* dependent Hfq peak within a GME spanning the *ybck*-gene. This sequence was selected for *in vitro* analysis. *Ppk*-dependent Hfq motifs (underlined and bolded to distinguish motifs that overlap) are indicated. **(B)** Results of Multiple Em for Motif Elicitation (MEME) finder for *ppk*-dependent Hfq motifs (brown), *ppk*-independent Hfq motifs (green), and secondary Hfq motifs (gold). The $\log_2$ motif enrichment was calculated across each category to demonstrate the specificity of the motif for its specified category. **(C)** Select *ppk*-dependent and non-Hfq binding DNA sequences used in this study.

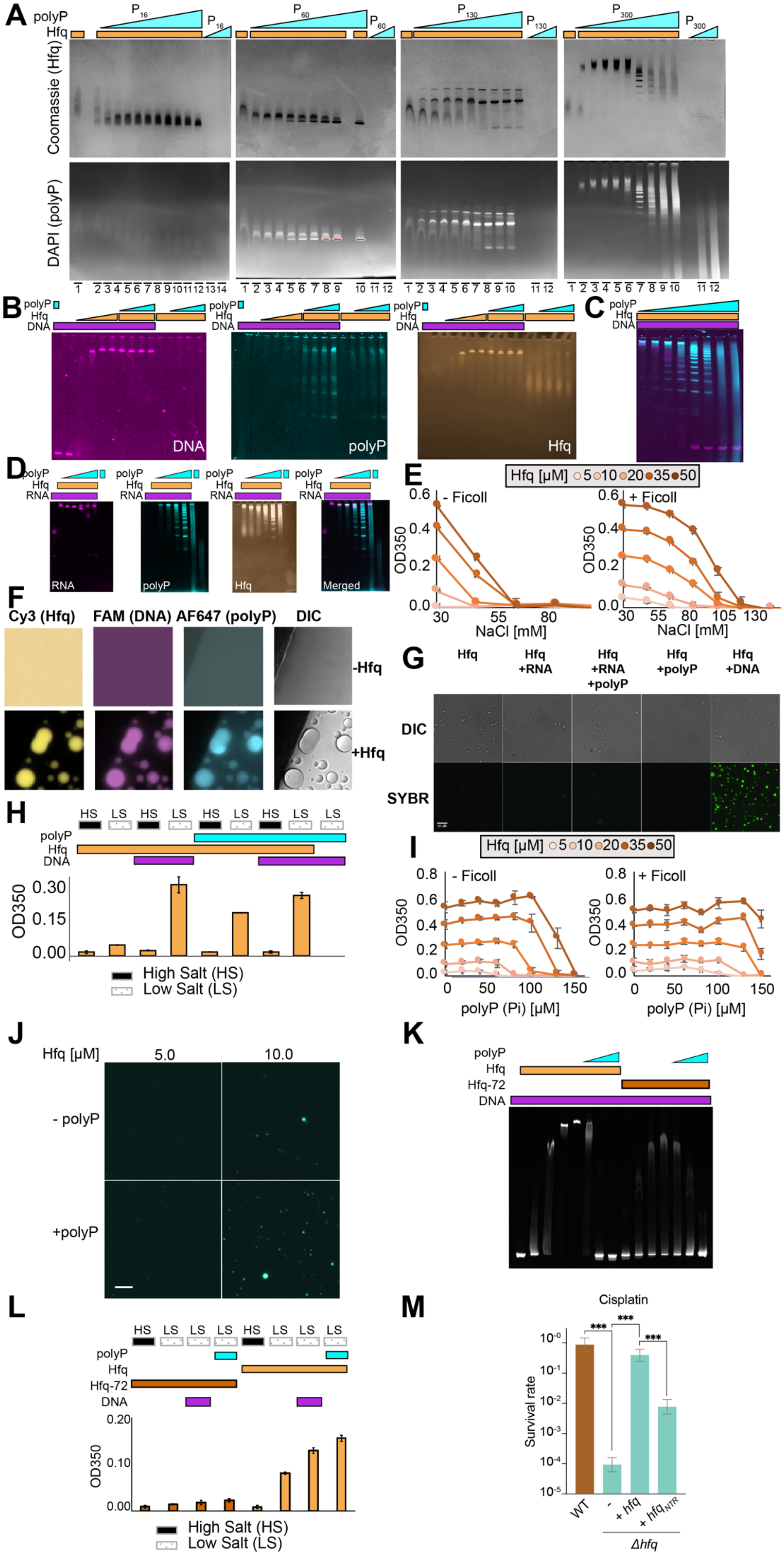
A
polyP
Hfq
Coomassie (Hfq)
DAPI (polyP)
B
DNA
polyP
Hfq
C
D
RNA
Merged
E
Hfq [µM] 5 10 20 35 50
- Ficoll
+ Ficoll
OD350
NaCl [mM]
F
Cy3 (Hfq)
FAM (DNA)
AF647 (polyP)
DIC
-Hfq
+Hfq
G
Hfq
Hfq +RNA
Hfq +RNA +polyP
Hfq +polyP
Hfq +DNA
SYBR
H
High Salt (HS)
Low Salt (LS)
I
polyP (Pi) [µM]
J
Hfq [µM]
5.0
10.0
- polyP
+polyP
K
Hfq-72
L
M
Cisplatin
Survival rate
WT
+ hfq
Δhfq

Fig. S4: *In vitro* polyP-Hfq-DNA interactions

(A) Mobility of Hfq (25 µM) on native gels without or with 25, 50, 62.5, 75, 100, 125, 250, 375 or 500 µM of polyP16, polyP60, polyP130, polyP300; polyP alone was analyzed at either 250 or 500 µM. Gels were stained with DAPI to visualize polyP (lower panel) followed by Coomassie staining to visualize Hfq (upper panel). **(B)** EMSA of FAM-labeled *ybck*-DNA (75 nM) with 30 µM AF647-polyP300 (lane 1), alone (lane 2) or with 5, 10, 17.5 or 25 µM Hfq (lanes 3-6). 0, 10, 15, 20 or 30 µM AF647-polyP300 (in Pi units) was added to 25 µM Hfq and DNA (lane 6-10) or Hfq alone (11-15). DNA and polyP were visualized using fluorescence imager. Gels were stained with Coomassie to detect Hfq. **(C)** EMSA of 70 nM *ybck*-DNA in the presence of 25 µM Hfq and increasing concentrations of polyP300 (20, 30, 50, 100, 150, 200, 300, 500 µM). **(D)** EMSA of 50 nM essD RNA in the presence of 25 µM Hfq and increasing concentrations of AF647-polyP300 (25, 50, 125, 250, 500 µM). Gel was stained with SYBR safe to visualize RNA, and AF647-polyP300 was visualized using a fluorescence imager. Gel was stained with Coomassie to detect Hfq. **(E)** Turbidity of different concentrations of Hfq (stored in 300 mM NaCl) upon 1:10 dilution into buffer containing the indicated concentrations of NaCl without or with 10% w/v ficoll70. The final Hfq concentrations are depicted in the figure. Turbidity at 350 nm was monitored. **(F)** Microscopic analysis of liquid droplet formation of 100 nM FAM-labelled DNA-fragment and 100 µM AF647-labelled polyP300 in the absence (upper panel) or presence (lower panel) of 100 µM Hfq, supplemented with 0.8 µM Cy3-labeled Hfq-S65C, under low salt conditions. **(G)** Microscopic analysis of liquid droplet formation of 50 µM Hfq in the presence or absence of 75 nM essD RNA, 75 nM *ppk*-dependent DNA and/or 50 µM polyP300 under low salt conditions. Samples were supplemented with 1:10,000 diluted SYBR Safe to visualize the nucleotides. **(H)** Turbidity of 500 µM Hfq (stored in 300 mM NaCl) upon 1:10 dilution into buffer containing either no additional salt (low salt) or 270 mM NaCl (high salt) in the absence or presence of 50 nM *ybck*-DNA and/or 15 µM polyP300. Turbidity at 350 nm was monitored. **(i)** Turbidity of different concentrations of Hfq (stored in 300 mM NaCl) upon 1:10 dilution into buffer containing the indicated concentrations of polyP$_{300}$ without or with 10% w/v ficoll-70. The final Hfq concentrations are depicted in the figure. Turbidity at 350 nm was monitored. All experiments in this figure were repeated at least 3 times and the mean +/- Std is shown. **(J).** Microscopic analysis of liquid droplet formation of 5 or 10 µM Hfq in the absence (upper panel) or presence (lower panel) of equimolar polyP$_{300}$, supplemented with 0.08 µM Cy3-labeled Hfq-S65C, under low salt conditions. Scale bar represents 10 µm. **(K)** EMSA of 75 nM *ybck*-DNA in the absence (lane 1) or presence of 5, 10, 17.5 or 25 µM Hfq (lanes 2-5) or Hfq-72 (lanes 9-12). 50, 125 or 250 µM of polyP300 was added to 25 µM Hfq (lanes 6-8) or Hfq-72 (lanes 13-15) and DNA. Gel was stained with SYBR Safe to visualize DNA. **(L)** Turbidity of 250 µM Hfq-72 or full-length Hfq (stored in 300 mM NaCl) upon 1:10 dilution into buffer containing either no additional salt (low salt) or 270 mM NaCl (high salt) in the absence or presence of 30 nM *ppk*-dependent DNA or 20 µM polyP300. Turbidity at 350 nm was monitored. **(M)** Survival of MG1655 wild-type or the *ppk* deletion strain expressing either hfq or hfq-72 from a plasmid in the presence of 4 µg/µl cisplatin. *** indicates a greater than 99.5% posterior probability that the indicated survival rates differ (using a Bayesian analysis of the survival data; see Methods for details).

Table S1. Strains, Plasmids and Oligos used in this study

Relevant Genotype	Markers	Source
BL21 (DE3) *(F- ompT gal dcm lon hsdSB(rB- mB-) λ(DE3 [lacI lacUV5-T7 gene 1 ind1 sam7 nin5]) ykgD::cat+)*		
MG1655 *(F-, λ-, rph-1 ilvG- rfb-50)*		(*43*)
MG1655 Δ*ppk*		(*3*)
MG1655 Δ*hfq*		this study
MG1655 Δ*hfq*Δ*ppk*		this study
MG1655 Δ*fis*		this study
MG1655 Δ*fis*Δ*ppk*		this study
MG1655 Δ*hns*		this study
MG1655 Δ*hns*Δ*ppk*		this study
MG1655 Δ*hupA*		this study
MG1655 Δ*hupA*Δ*ppk*		this study
MG1655 Δ*hupB*		this study
MG1655 Δ*hupB*Δ*ppk*		this study
MG1655 Δ*stpA*		this study
MG1655 Δ*stpA*Δ*ppk*		this study
MDS42		(*46*)
MDS42 Δ*ppk*		this study
MDS42 Δ*hfq*		this study
MG1655 pBAD18b	amp	(*3*)
MG1655 Δ*ppk* pBAD18b	amp	(*3*)
MG1655 pBAD18b-*ppk*	amp	(*3*)
MG1655 Δ*ppk* pBAD18b *ppk*	amp	(*3*)
MG1655 pBAD18b-hfq	amp	this study
MG1655 Δ*ppk* pBAD18b-*hfq*	amp	this study
MG1655 Δ*hfq* pBAD18b-*hfq*	amp	this study
MG1655 Δ*hfq* pBAD18b-*hfq*	amp	this study
MG1655 Δ*hfq* pBAD18b-*hfq-72*	amp	this study
MG1655 *hfq-PAmcherry*		this study
MG1655 *hfq-PAmcherry* Δ*ppk*		this study
BL21(DE3) pET21a-*hfq*	amp	this study
BL21(DE3) pET21a-*hfq*S65C	amp	this study
BL21(DE3) pET21a-*hfq-72*	amp	this study
MG1655 mal::lacIq ΔaraBAD lacI'-PBAD::cat-sacB::lacZ	cm	(*20*)
MG1655 mal::lacIq ΔaraBAD lacI'::PBAD-ompX-lacZ		(*20*)
MG1655 mal::lacIq ΔaraBAD lacI'::PBAD-ompX-lacZ Δhfq::cat-sacB	cm	(*20*)

MG1655 mal::lacIq ΔaraBAD lacI'::PBAD-rpoS-lacZ		(*20*)
MG1655 mal::lacIq ΔaraBAD lacI'::PBAD-rpoS-lacZ Δhfq::cat-sacB purA+	cm	(*20*)
MG1655 mal::lacIq ΔaraBAD lacI'::PBAD-flhD-lacZ		(*20*)
MG1655 mal::lacIq ΔaraBAD lacI'::PBAD-flhD-lacZ Δhfq::cat-sacB purA+	cm	(*20*)
MG1655 mal::lacIq ΔaraBAD lacI'::PBAD -chiP-lacZ		(*20*)
MG1655mal::lacIq ΔaraBAD lacI'::PBAD -chiP-lacZ Δhfq::cat-sacB purA+	cm	(*20*)
MG1655 mal::lacIq ΔaraBAD lacI':: PBAD -sodB-lacZ		(*20*)
MG1655 mal::lacIq ΔaraBAD lacI':: PBAD -sodB-lacZ Δhfq::cat-sacB purA+	cm	(*20*)
MG1655 mal::lacIq ΔaraBAD lacI'::PBAD-219sdhC-lacZ		(*9*)
MG1655 mal::lacIq ΔaraBAD lacI'::PBAD-219sdhC-lacZ Δhfq::cat-sacB purA+	cm	(*20*)
MG1655 mal::lacIq ΔaraBAD lacI'-P_{BAD}::cat-sacB::lacZ ppk::kan	cm kan	this study
MG1655 mal::lacIq ΔaraBAD lacI'::PBAD-ompX-lacZ ppk::kan	cm kan	this study
MG1655 mal::lacIq ΔaraBAD lacI'::PBAD-ompX-lacZ Δhfq::cat-sacB ppk::kan	cm kan	this study
MG1655 mal::lacIq ΔaraBAD lacI'::PBAD-rpoS-lacZ ppk::kan	cm kan	this study
MG1655 mal::lacIq ΔaraBAD lacI'::PBAD-rpoS-lacZ Δhfq::cat-sacB purA+ ppk::kan	cm kan	this study
MG1655 mal::lacIq ΔaraBAD lacI'::PBAD-flhD-lacZ ppk::kan	cm kan	this study
MG1655 mal::lacIq ΔaraBAD lacI'::PBAD-flhD-lacZ Δhfq::cat-sacB purA+ ppk::kan	cm kan	this study
MG1655 mal::lacIq ΔaraBAD lacI'::PBAD -chiP-lacZ ppk::kan	cm kan	this study
MG1655 mal::lacIq ΔaraBAD lacI'::PBAD -chiP-lacZ Δhfq::cat-sacB purA+ ppk::kan	cm kan	this study
MG1655 mal::lacIq ΔaraBAD lacI':: PBAD -sodB-lacZ ppk::kan	cm kan	this study
MG1655 mal::lacIq ΔaraBAD lacI':: PBAD -sodB-lacZ Δhfq::cat-sacB purA+ ppk::kan	cm kan	this study
MG1655 mal::lacIq ΔaraBAD lacI'::PBAD-219sdhC-lacZ ppk::kan	cm kan	this study
MG1655 mal::lacIq ΔaraBAD lacI'::PBAD-219sdhC-lacZ Δhfq::cat-sacB purA+ ppk::kan	cm kan	this study
MG1655 mal::lacIq ΔaraBAD lacI'-PBAD::cat-sacB::lacZ pBR-plac	amp	this study
MG1655 mal::lacIq ΔaraBAD lacI'::PBAD-rpoS-lacZ pBRplac-*dsrA*	amp	this study
MG1655 mal::lacIq ΔaraBAD lacI'::PBAD-rpoS-lacZ Δhfq::cat-sacB purA+ pBRplac-*dsrA*	amp cm	this study
MG1655 mal::lacIq ΔaraBAD lacI'::PBAD-rpoS-lacZ pBRplac-*arcZ*	amp	this study
MG1655 mal::lacIq ΔaraBAD lacI'::PBAD-rpoS-lacZ Δhfq::cat-sacB purA+ pBRplac-*arcZ*	amp cm	this study

MG1655 mal::lacIq ΔaraBAD lacI'::PBAD-flhD-lacZ pBRplac-arcZ		amp	this study
MG1655 mal::lacIq ΔaraBAD lacI'::PBAD-flhD-lacZ Δhfq::cat-sacB purA+ pBRplac-*arcZ*		amp cm	this study
MG1655 mal::lacIq ΔaraBAD lacI'::PBAD-flhD-lacZ pBRplac-McaS		amp	this study
MG1655 mal::lacIq ΔaraBAD lacI'::PBAD-flhD-lacZ Δhfq::cat-sacB purA+ pBRplac-McaS		amp cm	this study
MG1655 mal::lacIq ΔaraBAD lacI':: PBAD -sodB-lacZ pBRplac-RyhB		amp	this study
MG1655 mal::lacIq ΔaraBAD lacI':: PBAD -sodB-lacZ Δhfq::cat-sacB purA+ pBRplac-RyhB		amp cm	this study
MG1655 mal::lacIq ΔaraBAD lacI'::PBAD-219sdhC-lacZ pBRplac-RyhB		amp	this study
MG1655 mal::lacIq ΔaraBAD lacI'::PBAD-219sdhC-lacZ Δhfq::cat-sacB purA+ pBRplac-*ryhB*		amp cm	this study
MG1655 mal::lacIq ΔaraBAD lacI'-PBAD::cat-sacB::lacZ ppk::kan pBR-plac		amp kan	this study
MG1655 mal::lacIq ΔaraBAD lacI'::PBAD-rpoS-lacZ ppk::kan pBRplac-*dsrA*		amp kan	this study
MG1655 mal::lacIq ΔaraBAD lacI'::PBAD-rpoS-lacZ Δhfq::cat-sacB purA+ ppk::kan pBRplac-*dsrA*		amp cm kan	this study
MG1655 mal::lacIq ΔaraBAD lacI'::PBAD-rpoS-lacZ ppk::kan pBRplac-*arcZ*		amp kan	this study
MG1655 mal::lacIq ΔaraBAD lacI'::PBAD-rpoS-lacZ Δhfq::cat-sacB purA+ ppk::kan pBRplac-*arcZ*		amp cm kan	this study
MG1655 mal::lacIq ΔaraBAD lacI'::PBAD-flhD-lacZ ppk::kan pBRplac-*arcZ*		amp kan	this study
MG1655 mal::lacIq ΔaraBAD lacI'::PBAD-flhD-lacZ Δhfq::cat-sacB purA+ ppk::kan pBRplac-arcZ		amp cm kan	this study
MG1655 mal::lacIq ΔaraBAD lacI'::PBAD-flhD-lacZ ppk::kan pBRplac-*mcaS*		amp kan	this study
MG1655 mal::lacIq ΔaraBAD lacI'::PBAD-flhD-lacZ Δhfq::cat-sacB purA+ ppk::kan pBRplac-*mcaS*		amp cm kan	this study
MG1655 mal::lacIq ΔaraBAD lacI':: PBAD -sodB-lacZ ppk::kan pBRplac-*ryhB*		amp kan	this study
MG1655 mal::lacIq ΔaraBAD lacI':: PBAD -sodB-lacZ Δhfq::cat-sacB purA+ ppk::kan pBRplac-*ryhB*		amp cm kan	this study
MG1655 mal::lacIq ΔaraBAD lacI'::PBAD-219sdhC-lacZ ppk::kan pBRplac-*ryhB*		amp kan	this study
MG1655 mal::lacIq ΔaraBAD lacI'::PBAD-219sdhC-lacZ Δhfq::cat-sacB purA+ ppk::kan pBRplac-*ryhB*		amp cm kan	this study
**Plasmid**	**Description**	**Marker (s)**	**Source**
pBAD18b	cloning vector with PBAD arabinose-inducible promoter	amp	(*60*)
pKD46	λ Red recombinase	amp	(*44*)
pCP20	Flp recombinase	amp	(*44*)
pKD3	cat chloramphenicol resistance casette donor	cat	(*44*)
pKD4	Kan Kanamycin resistance casette donor	Kan	(*44*)
pet21a	IPTG inducible vector for protein purification	amp	Novagen

pBAD18b-*hfq*	*hfq* arabinose inducible expressing vector	amp	this study
pBAD18b-*hfq-72*	*Hfq-NTR (1-72)* arabinose inducible expressing vector	amp	this study
pBAD18b-*ppk*	*ppk* arabinose inducible expression vector	amp	(*3*)
pBR-plac	IPTG inducible empty expression vector	amp	(*20*)
pBRplac-*dsrA*	IPTG inducible *dsrA* sRNA expression vector	amp	(*20*)
pBRplac-*arcZ*	IPTG inducible *arcZ* sRNA expression vector	amp	(*20*)
pBRplac-*mcaS*	IPTG inducible *mcaS* sRNA expression vector	amp	(*20*)
pBRplac-*ryhB*	IPTG inducible *ryhB* sRNA expression vector	amp	(*20*)
pet21a-*hfq*	IPTG inducible *hfq* vector for protein purification	amp	this study
pET-21a-*hfqS65C*	IPTG inducible *hfqS65C* vector for protein purification	amp	this study
pet21a-*hfq-72*	IPTG inducible *hfq-72* vector for protein purification	amp	this study

**Oligonucleotides[2]**	**Sequence**
pBAD_F	CTGTTTCTCCATACCCGTT
pBAD_R	GGCTGAAAATCTTCTCTCAT
ppk_lambdared_F	ATGGGTCAGGAAAAGCTATACATCGAAAAAGAGCTCGTGTAG GCTGGAGCTGCTTC
ppk_lambdared_R	TTATTCAGGTTGTTCGAGTGATTTGATGTAGTCATACATATGA ATATCCTCCTTA
ppk_F	CGTAATTAAAGCGCCAGCTC
ppK_R	ATCTGCATGGCACCATCTAC
hfq_pet21_F_NdeI	GTGATCATATGGCTAAGGGGCAATCTTTACAAG
hfq_pet21_R_XhoI	GATACCTCGAGTTATTCGGTTTCTTCGCTGTCCTGTTGC
hupA_F	CTGATTTGTCGTACCTGGAG
hupA_R	GACTACAGGCAGTGAGAAGC
hupB_ F	TGTCTCGCTAAGTTAGATGG
hupB_R	CAATTGTCAGCCCACAAGAC
stpA_ F	GGAATTAGCGAGCAGAGAGC
stpA_R	TACTGTTTGCAGGAATCAGC
hfq_ F	GTATTACAGGTTGTTGGTGC
hfq_R	AGACCAGAGATTCAAACTCC
hfq_lambdared_F	ATGGCTAAGGGGCAATCTTTACAAGATCCGTTCCTGGTGTAG GCTGGAGCTGCTTC
hfq_lambdared_R	TTATTCGGTTTCTTCGCTGTCCTGTTGCGCGGAAGTCATATGA ATATCCTCCTTA
hns_ F	CTCAACAAACCACCCCAATA
hns_R	TGGCGGGATTTTAAGCAAGT
hns_lambdared_F	CCTCAACAAACCACCCCAATATAAGTTTGAGATTACTACAGT

	GTAGGCTGGAGCTGCTTC
hns_lambdared_R	GCCGCTGGCGGGATTTTAAGCAAGTGCAATCTACAAAAGACA TATGAATATCCTCCTTAG
fis_ F	GCACATTCAACGCCATTGAG
fis_R	GGTCACTCCCTTTGTGACAC
fis_lambdared_F	ATGTTCGAACAACGCGTAAATTCTGACGTACTGACCGTGTAG GCTGGAGCTGCTTC
fis_lambdared_R	TTAGTTCATGCCGTATTTTTTCAATTTTTTACGCAGCATATGAA TATCCTCCTTA
hfq_F_BamHI	TCGGATCCGCATATAAGGAAAAGAGAGA
hfq_R_HindIII	TCAAGCTTCCGAAACCTTATTCGGTTTC
cycA_ F	ACTCTGATGCCGGTAGGTTC
cycA_R	GCGCCATCCAGCATGATA
HEX-nohD_F	TCTCTAGAGAAAGGGATGCTGAAATTGAG
HEX-ipex_F	TCGAATTCAGGTTGTGCTTCTAAAGGAAG
HEX-cynR-cynT_F	TCGAATTCGGTGAAGCTGCCATGTTCAG
cynR-cynT_R	TCGGTACCGCTGTTTAAACAAGGCTTCC
ipex_R	TCGGTACCCACCTTCCCTAAAGCACTCG
nohD_R	TCAAGCTTCATTCACCTCACGGATGTAG
Yfck_F	GCATGCACAATCAGGAGCTT
YfcK_R	CAATGAGCGCCTTTCCATCC
Ppk-dependent_F	GCAATCAGCCAAAGCCTGAG
Ppk-dependent_R	AAAGGGACAGGCATCAAGGG
Non-binding site_F	CAGGATTACGCCGACTGGAT
Non-binding site _R	TCGCGGCTAACTCCTGATTC
Hfq S65C_F	CGATTTCTACTGTTGTCCCGTGTCGCCCGGTTT
Hfq S65C_R	AAACCGGGCGACACGGGACAACAGTAGAAATCG
EssD_T7-F	TAATACGACTCACTATAGATAAATATTCATCTAATCAATGTGA TTAT
EssD_T7-R	CACCCGTTGTTAACTTATC

[1]:amp, ampicillin; kan, Kanamycin; cm, chloramphenicol. [2]: F: forward primer; R: reverse primer.

Movie S1. Liquid-liquid phase separation of 50 µM purified Hfq in 25 mM Tris, 30 mM NaCl, pH 7.5.

Movie S2. Liquid-liquid phase separation of 100 µM purified Hfq in 25 mM Tris, 30 mM NaCl, pH 7.5.

Movie S3. Liquid-liquid phase separation of 400 µM purified Hfq in 25 mM Tris, 30 mM NaCl, pH 7.5.

Movie S4. Fluorescence recovery upon photobleaching of 100 µM purified Hfq supplemented with 0.8 µM Cy3-labeled Hfq-S65C in 25 mM Tris, 30 mM NaCl, pH 7.5.

Movie S5. Fluorescence recovery upon photobleaching of 100 µM purified Hfq supplemented with 0.8 µM Cy3-labeled Hfq-S65C in the presence of 100 nM FAM-labeled DNA fragment in 25 mM Tris, 30 mM NaCl, pH 7.5.

Movie S6. Fluorescence recovery upon photobleaching of 100 µM purified Hfq supplemented with 0.8 µM Cy3-labeled Hfq-S65C in the presence of 100 µM AF647-labeled $polyP_{300}$ in 25 mM Tris, 30 mM NaCl, pH 7.5.

Movie S7. Fluorescence recovery upon photobleaching of 100 µM purified Hfq supplemented with 0.8 µM Cy3-labeled Hfq-S65C and 100 µM AF647-labeled $polyP_{300}$ in 25 mM Tris, 30 mM NaCl, pH 7.5.

Data S1. Locations of all prophages and mobile genetic elements (MGEs) as used in the present analysis, defined based on data from Ecocyc (*27*).

Data S2. Locations of EPODs in wild type MG1655 during growth in M9/RDM/glucose media.

REFERENCES AND NOTES

1. M. R. Brown, A. Kornberg, Inorganic polyphosphate in the origin and survival of species. *Proc. Natl. Acad. Sci. U.S.A.* **101**, 16085–16087 (2004).

2. L. Xie, A. Rajpurkar, E. Quarles, N. Taube, A. S. Rai, J. Erba, B. Sliwinski, M. Markowitz, U. Jakob, D. Knoefler, Accumulation of nucleolar inorganic polyphosphate is a cellular response to cisplatin-induced apoptosis. *Front. Oncol.* **9**, 1410 (2019).

3. M. J. Gray, W. Y. Wholey, N. O. Wagner, C. M. Cremers, A. Mueller-Schickert, N. T. Hock, A. G. Krieger, E. M. Smith, R. A. Bender, J. C. A. Bardwell, U. Jakob, Polyphosphate is a primordial chaperone. *Mol. Cell* **53**, 689–699 (2014).

4. F. Beaufay, E. Quarles, A. Franz, O. Katamanin, W.-Y. Wholey, U. Jakob, Polyphosphate functions in vivo as an iron chelator and fenton reaction inhibitor. *MBio* **11**, 4 (2020).

5. A. Du Toit, Phage induction in different contexts. *Nat. Rev. Microbiol.* **17**, 126–127 (2019).

6. G. Pósfai, G. Plunkett III, T. Fehér, D. Frisch, G. M. Keil, K. Umenhoffer, V. Kolisnychenko, B. Stahl, S. S. Sharma, M. de Arruda, V. Burland, S. W. Harcum, F. R. Blattner, Emergent properties of reduced-genome *Escherichia coli*. *Science* **312**, 1044–1046 (2006).

7. R. P. Rastogi, Richa, A. Kumar, M. B. Tyagi, R. P. Sinha, Molecular mechanisms of ultraviolet radiation-induced DNA damage and repair. *J. Nucleic Acids* **2010**, 592980 (2010).

8. T. Feher, B. Cseh, K. Umenhoffer, I. Karcagi, G. Posfai, Characterization of cycA mutants of *Escherichia coli*. An assay for measuring in vivo mutation rates. *Mutat. Res.* **595**, 184–190 (2006).

9. D. J. Jin, C. A. Gross, Mapping and sequencing of mutations in the *Escherichia coli* rpoB gene that lead to rifampicin resistance. *J. Mol. Biol.* **202**, 45–58 (1988).

10. N. N. Rao, M. R. Gomez-Garcia, A. Kornberg, Inorganic polyphosphate: Essential for growth and survival. *Annu. Rev. Biochem.* **78**, 605–647 (2009).

11. K. Murata, S. Hagiwara, Y. Kimori, Y. Kaneko, Ultrastructure of compacted DNA in cyanobacteria by high-voltage cryo-electron tomography. *Sci. Rep.* **6**, 34934 (2016).

12. R. T. Dame, The role of nucleoid-associated proteins in the organization and compaction of bacterial chromatin. *Mol. Microbiol.* **56**, 858–870 (2005).

13. J. McQuail, A. Switzer, L. Burchell, S. Wigneshweraraj, The RNA-binding protein Hfq assembles into foci-like structures in nitrogen starved *Escherichia coli*. *J. Biol. Chem.* **295**, 12355–12367 (2020).

14. I. Moll, D. Leitsch, T. Steinhauser, U. Blasi, RNA chaperone activity of the Sm-like Hfq protein. *EMBO Rep.* **4**, 284–289 (2003).

15. K. M. Wassarman, F. Repoila, C. Rosenow, G. Storz, S. Gottesman, Identification of novel small RNAs using comparative genomics and microarrays. *Genes Dev.* **15**, 1637–1651 (2001).

16. J. Orans, A. R. Kovach, K. E. Hoff, N. M. Horstmann, R. G. Brennan, Crystal structure of an *Escherichia coli* Hfq Core (residues 2-69)-DNA complex reveals multifunctional nucleic acid binding sites. *Nucleic Acids Res.* **48**, 3987–3997 (2020).

17. A. Takada, M. Wachi, A. Kaidow, M. Takamura, K. Nagai, DNA binding properties of the hfq gene product of *Escherichia coli*. *Biochem. Biophys. Res. Commun.* **236**, 576–579 (1997).

18. M. Kajitani, A. Kato, A. Wada, Y. Inokuchi, A. Ishihama, Regulation of the *Escherichia coli* hfq gene encoding the host factor for phage Q beta. *J. Bacteriol.* **176**, 531–534 (1994).

19. G. W. Li, D. Burkhardt, C. Gross, J. S. Weissman, Quantifying absolute protein synthesis rates reveals principles underlying allocation of cellular resources. *Cell* **157**, 624–635 (2014).

20. A. Zhang, D. J. Schu, B. C. Tjaden, G. Storz, S. Gottesman, Mutations in interaction surfaces differentially impact E. coli Hfq association with small RNAs and their mRNA targets. *J. Mol. Biol.* **425**, 3678–3697 (2013).

21. T. Vora, A. K. Hottes, S. Tavazoie, Protein occupancy landscape of a bacterial genome. *Mol. Cell* **35**, 247–253 (2009).

22. P. L. Freddolino, H. M. Amemiya, T. J. Goss, S. Tavazoie, Dynamic landscape of protein occupancy across the *Escherichia coli* chromosome. *PLoS Biol.* **19**, e3001306 (2021).

23. H.M. Amemiya, T.J. Goss, T.M. Nye, R. Hurto, L. A.Simmons, P.L.Freddolino, Distinct heterochromatin-like domains promote transcriptional memory and silence parasitic genetic elements in bacteria. *EMBO J.*, in press.

24. C. Sauter, J. Basquin, D. Suck, Sm-like proteins in Eubacteria: The crystal structure of the Hfq protein from *Escherichia coli*. *Nucleic Acids Res.* **31**, 4091–4098 (2003).

25. S. Melamed, A. Peer, R. Faigenbaum-Romm, Y. E. Gatt, N. Reiss, A. Bar, Y. Altuvia, L. Argaman, H. Margalit, Global mapping of small RNA-target interactions in bacteria. *Mol. Cell* **63**, 884–897 (2016).

26. A. E. Posey, A. S. Holehouse, R. V. Pappu, Phase separation of intrinsically disordered proteins. *Methods Enzymol.* **611**, 1–30 (2018).

27. F. Erdel, K. Rippe, Formation of chromatin subcompartments by phase separation. *Biophys. J.* **114**, 2262–2270 (2018).

28. T. Ali Azam, A. Iwata, A. Nishimura, S. Ueda, A. Ishihama, Growth phase-dependent variation in protein composition of the *Escherichia coli* nucleoid. *J. Bacteriol.* **181**, 6361–6370 (1999).

29. T. B. Updegrove, J. J. Correia, R. Galletto, W. Bujalowski, R. M. Wartell, E. coli DNA associated with isolated Hfq interacts with Hfq's distal surface and C-terminal domain. *Biochim. Biophys. Acta* **1799**, 588–596 (2010).

30. T. B. Updegrove, A. Zhang, G. Storz, Hfq: The flexible RNA matchmaker. *Curr. Opin. Microbiol.* **30**, 133–138 (2016).

31. S. Panja, S. A. Woodson, Fluorescence reporters for Hfq oligomerization and RNA annealing. *Methods Mol. Biol.* **1259**, 369–383 (2015).

32. H. H. Tuson, J. S. Biteen, Unveiling the inner workings of live bacteria using super-resolution microscopy. *Anal. Chem.* **87**, 42–63 (2015).

33. A. S. Hansen, M. Woringer, J. B. Grimm, L. D. Lavis, R. Tjian, X. Darzacq, Robust model-based analysis of single-particle tracking experiments with Spot-On. *eLife* **7**, e33125 (2018).

34. M. Yousuf, I. Iuliani, R. T. Veetil, A. S. N. Seshasayee, B. Sclavi, M. C. Lagomarsino, Early fate of exogenous promoters in E. coli. *Nucleic Acids Res.* **48**, 2348–2356 (2020).

35. W. W. Navarre, S. Porwollik, Y. Wang, M. McClelland, H. Rosen, S. J. Libby, F. C. Fang, Selective silencing of foreign DNA with low GC content by the H-NS protein in Salmonella. *Science* **313**, 236–238 (2006).

36. B. A. Shen, R. Landick, Transcription of bacterial chromatin. *J. Mol. Biol.* **431**, 4040–4066 (2019).

37. K. Singh, J. N. Milstein, W. W. Navarre, Xenogeneic silencing and its impact on bacterial genomes. *Annu. Rev. Microbiol.* **70**, 199–213 (2016).

38. T. A. Azam, A. Ishihama, Twelve species of the nucleoid-associated protein from *Escherichia coli*. Sequence recognition specificity and DNA binding affinity. *J. Biol. Chem.* **274**, 33105–33113 (1999).

39. A. Malabirade, K. Jiang, K. Kubiak, A. Diaz-Mendoza, F. Liu, J. A. van Kan, J. F. Berret, V. Arluison, J. R. C. van der Maarel, Compaction and condensation of DNA mediated by the C-terminal domain of Hfq. *Nucleic Acids Res.* **45**, 7299–7308 (2017).

40. E. Fortas, F. Piccirilli, A. Malabirade, V. Militello, S. Trépout, S. Marco, A. Taghbalout, V. Arluison, New insight into the structure and function of Hfq C-terminus. *Biosci. Rep.* **35**, e00190 (2015).

41. A. G. Larson, D. Elnatan, M. M. Keenen, M. J. Trnka, J. B. Johnston, A. L. Burlingame, D. A. Agard, S. Redding, G. J. Narlikar, Liquid droplet formation by HP1α suggests a role for phase separation in heterochromatin. *Nature* **547**, 236–240 (2017).

42. A. R. Strom, A. V. Emelyanov, M. Mir, D. V. Fyodorov, X. Darzacq, G. H. Karpen, Phase separation drives heterochromatin domain formation. *Nature* **547**, 241–245 (2017).

43. F. R. Blattner, G. Plunkett III, C. A. Bloch, N. T. Perna, V. Burland, M. Riley, J. Collado-Vides, J. D. Glasner, C. K. Rode, G. F. Mayhew, J. Gregor, N. W. Davis, H. A. Kirkpatrick, M. A. Goeden, D. J. Rose, B. Mau, Y. Shao, The complete genome sequence of *Escherichia coli* K-12. *Science* **277**, 1453–1462 (1997).

44. K. A. Datsenko, B. L. Wanner, One-step inactivation of chromosomal genes in *Escherichia coli* K-12 using PCR products. *Proc. Natl. Acad. Sci. U.S.A.* **97**, 6640–6645 (2000).

45. J. Salvatier, Thomas V. Wiecki, C. Fonnesbeck, Probabilistic programming in Python using PyMC3. *Peer J. Comput. Sci.* **2**, e55 (2016).

46. A. Ducret, E. M. Quardokus, Y. V. Brun, MicrobeJ, a tool for high throughput bacterial cell detection and quantitative analysis. *Nat. Microbiol.* **1**, 16077 (2016).

47. E. Laskowska, J. Bohdanowicz, D. Kuczyńska-Wiśnik, E. Matuszewska, S. Kędzierska, A. Taylor, Aggregation of heat-shock-denatured, endogenous proteins and distribution of the IbpA/B and Fda marker-proteins in *Escherichia coli* WT and grpE280 cells. *Microbiology* **150**, 247–259 (2004).

48. J. Miller, *A Short Course in Bacterial Genetics–A Laboratory Manual and Handbook for Escherichia coli and Related Bacteria* (Cold Spring Harbor Laboratory Press, 1992).

49. N. L. Bray, H. Pimentel, P. Melsted, L. Pachter, Near-optimal probabilistic RNA-seq quantification. *Nat. Biotechnol.* **34**, 525–527 (2016).

50. H. Pimentel, N. L. Bray, S. Puente, P. Melsted, L. Pachter, Differential analysis of RNA-seq incorporating quantification uncertainty. *Nat. Methods* **14**, 687–690 (2017).

51. C. R. Harris, K. J. Millman, S. J. van der Walt, R. Gommers, P. Virtanen, D. Cournapeau, E. Wieser, J. Taylor, S. Berg, N. J. Smith, R. Kern, M. Picus, S. Hoyer, M. H. van Kerkwijk, M. Brett, A. Haldane, J. F. del Río, M. Wiebe, P. Peterson, P. Gérard-Marchant, K. Sheppard, T. Reddy, W. Weckesser, H. Abbasi, C. Gohlke, T. E. Oliphant, Array programming with NumPy. *Nature* **585**, 357–362 (2020).

52. H. Wickham, *ggplot2 - Elegant Graphics for Data Analysis* (Springer, 2009).

53. B. Demeler, G. E. Gorbet, Analytical ultracentrifugation data analysis with UltraScan-III, in *Analytical Ultracentrifugation*, S. Uchiyama, F. Arisaka, W. F. Stafford, T. Laue, Eds. (Springer, 2016), pp. 119–143.

54. B. Demeler, E. Brookes, L. Nagel-Steger, Analysis of heterogeneity in molecular weight and shape by analytical ultracentrifugation using parallel distributed computing. *Methods Enzymol.* **454**, 87–113 (2009).

55. E. Brookes, W. Cao, B. Demeler, A two-dimensional spectrum analysis for sedimentation velocity experiments of mixtures with heterogeneity in molecular weight and shape. *Eur. Biophys. J.* **39**, 405–414 (2010).

56. J. Lempart, E. Tse, J. A. Lauer, M. I. Ivanova, A. Sutter, N. Yoo, P. Huettemann, D. Southworth, U. Jakob, Mechanistic insights into the protective roles of polyphosphate against amyloid cytotoxicity. *Life Sci. Alliance* **2**, e201900486 (2019).

57. S. A. Smith, J. H. Morrissey, Sensitive fluorescence detection of polyphosphate in polyacrylamide gels using 4′,6-diamidino-2-phenylindol. *Electrophoresis* **28**, 3461–3465 (2007).

58. C. Wong, S. Sridhara, J. C. Bardwell, U. Jakob, Heating greatly speeds Coomassie blue staining and destaining. *Biotechniques* **28**, 426–428, 430, 432 (2000).

59. B. P. Isaacoff, Y. Li, S. A. Lee, J. S. Biteen, SMALL-LABS: Measuring single-molecule intensity and position in obscuring backgrounds. *Biophys. J.* **116**, 975–982 (2019).

60. L. M. Guzman, D. Belin, M. J. Carson, J. Beckwith, Tight regulation, modulation, and high-level expression by vectors containing the arabinose PBAD promoter. *J. Bacteriol.* **177**, 4121–4130 (1995).
